# Silicon Nanowire Mats Enable Advanced Bioelectrical Recordings in Primary DRG Cell Cultures

**DOI:** 10.1002/adhm.202500379

**Published:** 2025-05-24

**Authors:** Ivano Lucarini, Francesco Maita, Giorgia Conte, Emanuela Saracino, Francesco Formaggio, Elena Palmieri, Roberta Fabbri, Aikaterini Konstantoulaki, Chiara Lazzarini, Marco Caprini, Valentina Benfenati, Luca Maiolo, Annalisa Convertino

**Affiliations:** ^1^ Institute for Microelectronics and Microsystems National Research Council, Via Fosso del Cavaliere 100 Rome 00133 Italy; ^2^ Institute for Organic Synthesis and Photoreactivity National Research Council, Via Gobetti 101 Bologna 40129 Italy; ^3^ Department of Pharmacy and Biotechnology University of Bologna via San Donato 19/2 Bologna 40127 Italy

**Keywords:** astrocytes, dorsal root ganglion neuron and glial cell cultures, intracellular recording, microelectrode arrays, silicon nanowires

## Abstract

Primary dorsal root ganglion (DRG) cell cultures provide a valuable model for studying in vitro sensory transduction, neuropathies, and chronic pain, as they replicate the in vivo heterogeneity of DRG neurons and non‐neuronal cells. However, traditional patch‐clamp techniques are invasive and cannot capture the collective cell dynamics. While planar multielectrode arrays (MEAs) offer a non‐invasive alternative, they suffer from poor cell‐electrode coupling and limited resolution for identifying specific DRG neuronal types like C‐fiber nociceptors, key targets in chronic pain research. This work demonstrates that silicon nanowire (SiNW) mat‐based MEAs, while maintaining their reduced invasiveness, enable continuous intracellular recordings from neurons in primary rat DRG cell cultures. Supported by a cortical astrocyte feeder layer, SiNW mats promote DRG neuron and glial cell growth preserving cells’ in vivo morphological and functional characteristics. Integrated into a compartmentalized MEA, they enable reliable recordings of drug‐modulated neuronal activity alongside a baseline related to the astrocyte layer. The recorded signals exhibit characteristics of intracellular action potentials, suggesting spontaneous intracellular access by SiNWs. Distinct electrophysiological signatures allow identifying C‐fiber nociceptors, as confirmed by patch‐clamp measurements. This platform represents a powerful tool for investigating in vitro pain mechanisms, with potential applications in preclinical pain research and pharmacological translational studies.

## Graphical abstract

SiNW mat‐based MEA (NW_MEA) enables continuous intracellular recordings from neurons in primary rat DRG cell cultures, identifying C‐fiber nociceptors. Supported by an astrocyte feeder layer, SiNW mat promotes DRG neuron and glial cell growth, preserving in vivo‐like cell characteristics and allowing drug‐modulated activity monitoring. NW_MEA provides a novel platform for in vitro pain research, opening new avenues for preclinical and translational pharmacological studies.

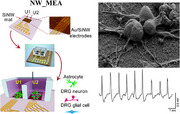

## 1 Introduction

Dorsal root ganglion (DRG) neurons are the primary transducers in the sensory pathway. They detect and convert various external stimuli, both innocuous and noxious, into electrochemical signals that are conveyed to and processed in the central nervous system (CNS).^[^ ^1^, ^2^, ^3^, ^4^, ^5^ ^]^ These sensory neurons form a heterogeneous and complex population, with different types and subtypes specialized in sensing thermal, mechanical, or chemical stimuli.^[^ ^3^, ^6^, ^7^ ^]^ Their cell bodies cluster within the DRG, while their bifurcated axons extend one branch into the peripheral nervous system (PNS) and the other one into CNS. In addition to the cell bodies of the sensory neurons, the DRGs also contain non‐neuronal cells, including glial cells (e.g., satellite and Schwann cells), immune cells, and vascular cells, all of which play essential roles in supporting the ganglia's function. Culturing primary DRG cells preserves this heterogeneity and complexity of cellular population, providing an effective model for studying sensory transduction mechanisms, neuropathies, nerve injury, and chronic pain.^[^ ^8^, ^9^, ^10^, ^11^, ^12^ ^]^ However, bioelectrical studies of DRG neurons typically rely on single‐cell patch‐clamp recordings. While invaluable for defining electrophysiological properties of any cell, these methods are invasive, unsuited for long‐term studies, and fail to capture the ensemble dynamics of neurons and glial cells. This limitation is particularly relevant given the increasing recognition that DRG glial cells may play a critical role in PNS pathologies, especially chronic pain.^[^ ^13^, ^14^, ^15^, ^16^ ^]^ Therefore, there is a growing need for methods, which enable the recording of bioelectrical activity in DRG neuron populations interacting with the glial cells, to advance our understanding of pain mechanisms and the development of new therapeutic treatments.

Multielectrode array (MEA) devices are well‐suited for non‐invasive and long‐term recording of the bioelectrical activity generated by ensembles of electrogenic cells, both in vitro and in vivo.^[^ ^17^, ^18^, ^19^ ^]^ They enable insights into collective dynamics, synchronization, and communication among cell to cell. Current studies on electrical signaling in DRG cell cultures predominantly leverage planar metallic or complementary metal‐oxide semiconductor (CMOS) MEAs, but these approaches face significant limitations. In particular, the poor electrical coupling between neurons and electrodes^[^ ^19^ ^]^ result in low‐amplitude signals (tens to hundreds of microvolts)^[^ ^20^, ^21^, ^22^, ^23^, ^24^, ^25^, ^26^, ^27^ ^]^ and a low signal‐to‐noise ratio, requiring a significant computational resource to extract data and sort out the recorded signals. Planar MEAs also fail to provide the richness of bioelectrical information obtained by patch‐clamp recordings, such as peculiar features in action potential’ (AP) shapes, which are crucial for identifying specific types of sensory neurons. For instance, C‐fiber nociceptor neurons, key targets in chronic pain research,^[^ ^28^, ^29^ ^]^ exhibit a “shoulder” in the falling phase of their APs that is the result of specific ion channel dynamics.^[^ ^6^, ^7^, ^30^ ^]^ This electrophysiological feature, combined with the small cell body diameter (10–30 µm) and the responsiveness to capsaicin (CAPS, an agonist of the channel transient receptor potential vanilloid 1, TRPV1),^[^ ^31^, ^32^ ^]^ is commonly used to identify C‐fiber nociceptors. Planar MEAs lack the resolution to detect the above mentioned electrical signatures, thus limiting their application in pain research. Recently, nanostructured electrodes, whose surfaces feature nanopillars or nanowires, have improved MEA sensitivity by enhancing cell‐electrode coupling and increasing the sensing area.^[^ ^19^, ^33^, ^34^, ^35^, ^36^, ^37^ ^]^ These nanostructures enable mechanisms like cellular engulfment or intracellular access, which improve signal quality. While successful in monocultures of cortical neurons or cardiomyocytes, their application to complex DRG cell cultures, including both neurons and glial cells, remains up to now largely unexplored. A notable exception is the U‐shaped nanowire field‐effect transistor (U‐NWFET),^[^ ^38^ ^]^ which can record full‐amplitude intracellular APs of single DRG neurons, resolving the characteristic shoulder feature of their AP shapes, similarly to patch‐clamp recordings. However, recording stability limited to a few tens of seconds, along with the high complexity of their design and fabrication process, hinders their scalability for studies requiring long‐term and simultaneous monitoring of multiple neurons.

Here we address the limitations in the electrophysiological study of the DRG cell cultures with MEA technology by leveraging the excellent cell‐interfacing properties and effective signal‐recording capabilities of silicon nanowire (SiNW) mats.^[^ ^39^, ^40^, ^41^, ^42^, ^43^, ^44^, ^45^, ^46^, ^47^ ^]^ Assisted by rat cortical astrocytes serving as feeder layer, SiNW mats promote the DRG neuron adhesion and neurite outgrowth as well as the spreading of peripheral glial cells, creating a supportive environment for cell growth and interaction. Integrated into MEAs, the SiNW mats enable reliable recording of drug‐modulated electrical activity from the neuronal component within the DRG cell culture. Notably, the recorded signals closely resemble the AP characteristics of the C‐fiber nociceptors, particularly by detecting the presence of a shoulder in the falling phase of the signal. This similarity is supported by patch‐clamp analysis, suggesting that the SiNWs allow intracellular access without the need of the typical techniques such as electroporation,^[^ ^48^, ^49^ ^]^ optoporation,^[^ ^50^, ^51^ ^]^ or mechanical insertion facilitated by phospholipid‐surface modification of the free standing probes,^[^ ^52^ ^]^ commonly used in most of the reported nanostructured MEA. Due to the rapid self‐healing of the cell membrane, these methods typically permit intracellular access for only tens of seconds, and the external stimulus can be applied only briefly without significantly perturbing the system. Consequently, these approaches restrict observations to pre‐selected cells at specific time points, limiting recordings to short durations rather than enabling continuous, unbiased monitoring of collective cellular dynamics.

Furthermore, intracellular recordings have been consistently obtained with NW_MEA over extended periods from different devices involving specimens from distinct animals, demonstrating the robustness and high sensitivity of the proposed approach.

Finally, we underscore that the integration of astrocytes in the DRG cell culture increases the model's complexity and physiological relevance. This model provides an effective platform to investigate the communication signaling between the CNS and PNS, particularly in the context of pain signal processing. Indeed, growing evidence highlights also the critical role of astrocytes in pain signal processing within the brain, suggesting that also their dysfunction (astrogliopathy) can contribute to chronic pain pathophysiology.^[^ ^53^, ^54^, ^55^ ^]^ By combining the physiological relevance of this PNS/CNS co‐culture model with the high sensitivity of SiNW mat‐based MEAs, our approach offers a powerful tool not only for basic research into pain mechanisms but also for translational studies. Specifically, it could be used to identify and validate novel therapeutic targets, such as neuron‐glia signaling pathways, and to screen potential drug candidates targeting DRG neuron and astrocyte interactions.

## 2 Results

### 2.1 Primary Rat DRG Neuron‐Glial Cell Culture on SiNW Mats: Growth and Population Characterization

#### 2.1.1 DRG Neuron‐Glial Cell Culture and Astrocyte Feeder Layer

We initially investigated whether SiNW mats alone could support DRG cell growth and maturation. The tested substrates included SiNW mats grown via plasma enhanced chemical vapor deposition (PECVD)^[^ ^40^, ^42^, ^46^ ^]^ on glass slides and coated with a Ti (20 nm)/Au (125 nm) bilayer (Au/SiNW mats) in order to reproduce the electrode surface characteristics of the developed MEA (Section 2.2). Scanning electron microscopy (SEM) image of the as‐grown SiNWs (Figure S1a, Supporting Information) reveals a dense mat of randomly arranged and oriented nanowires, 2–3 µm in length with base diameters of 50–80 nm. After metal deposition, the Au/SiNWs (Figure S1b, Supporting Information) exhibit cylindrical shapes with increased base diameters (120–180 nm) and unchanged lengths.

DRG cells, isolated from P11‐P21 rats, were directly cultured on the Au/SiNW mats following protocols detailed in Experimental Section. Molecular and morphological analyses (Figure S2, Supporting Information) demonstrated that the nanostructured platform alone did not support DRG neuronal and glial development. This is not surprising because primary neurons frequently require specific adhesion cues to attach on inorganic surfaces. To address this issue while ensuring direct neuron‐nanowire interaction, we avoided functionalizing the Au/SiNW surface with adhesion molecules. Instead, we cultured DRG cells with the assistance of an astrocyte feeder layer, leveraging astrocytes' well‐known neuro‐supportive properties.^[^ ^56^, ^57^ ^]^ Additionally, our previous studies^[^ ^46^ ^]^ demonstrated that astrocytes, plated directly on the Au/SiNW substrate, adhered to the nanowires without eliciting gliotic reactions, thus supporting neuronal development. After 5 days in vitro (DIV), DRG cells were seeded above the astrocyte layer (protocols in Experimental Section).

#### 2.1.2 Cell Viability and Morphology

Viability analysis of the cultures plated on both Au/SiNW and control (CTRL) substrates with the support of the astrocyte feeder layer was performed using FDA at 3 DIV and 7 DIV from the DRG cell plating. As CTRL, we utilized a planar Si substrate covered by the same Ti/Au bilayer as the SiNW mat. FDA images showed a variety of viable cells, with different morphologies, adhering to the substrates (Figure S3a‐d, Supporting Information). The quantitative analysis showed that both substrates demonstrated similar trends in cell growth over time, with an increase in cell number from 3 DIV to 7 DIV, confirming that Au/SiNWs support adhesion, proliferation, and growth of DRG cell populations.

**Figure 1:**
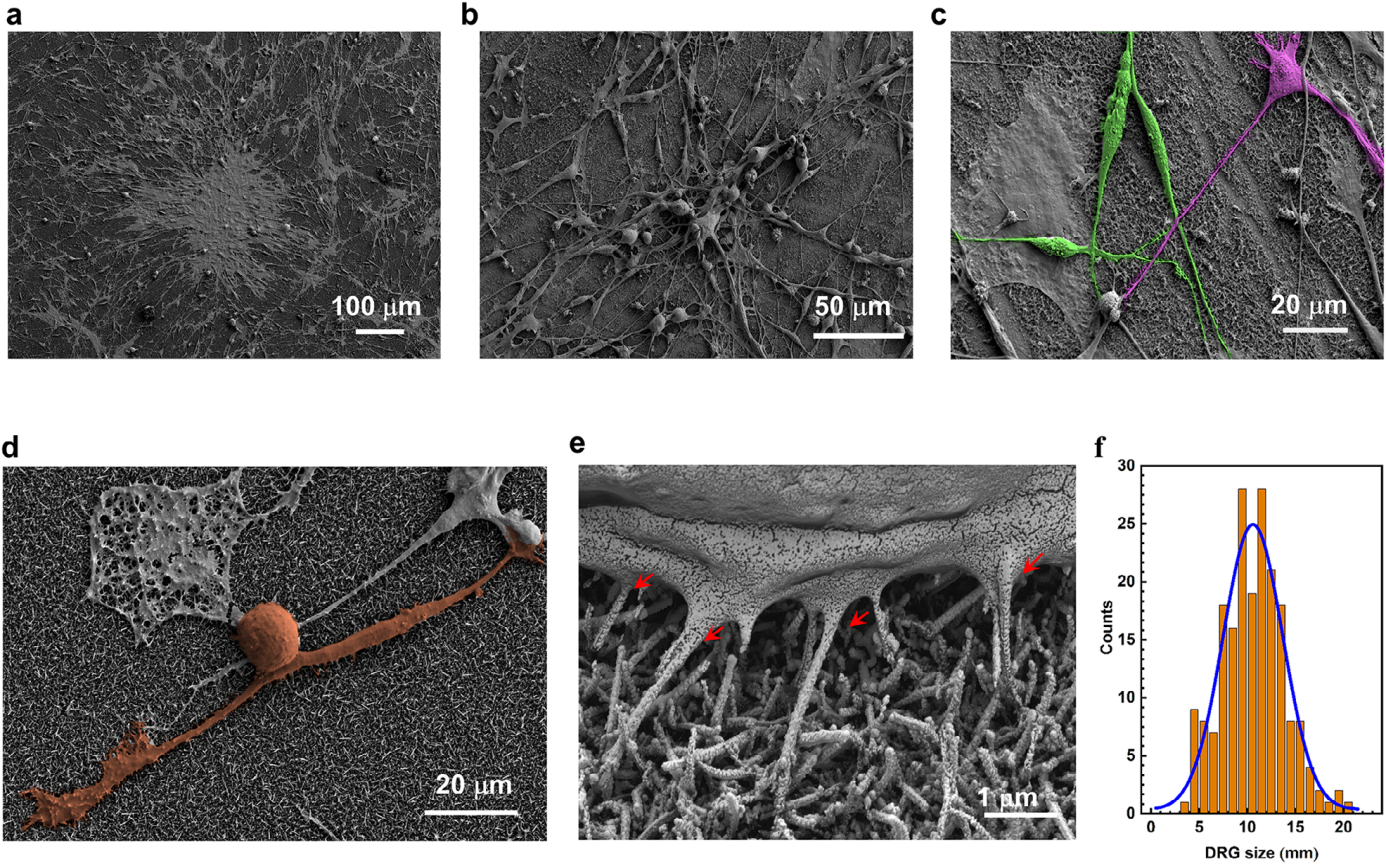
SEM characterization of the astrocyte/DRG cell culture on Au/SiNW substrate. a) Images showing the DRG cells above astrocyte feeder layer, plated on Au/SiNWs. The underlying large and lighter spots can be attributed to astrocytes. b) SEM image reveals cells with distinctive features, including c) cells showing spindle shaped body, and d) cells with a rounded soma and bifurcated projection resembling DRG glial cells and neurons, respectively. e) High magnification image taken at the tilted angle of 30° shows the nanowires below the cell body. f) Size distribution representation of the DRG neuron‐like cells in the samples and Gaussian fit of the data.

To gain deeper insights into the characteristics of the astrocyte/DRG cell culture, morphological investigations by SEM were performed on fixed cells after 5 DIV from the DRG cell plating on the astrocyte feeder layer. We can observe distinct morphological cellular features in **Figure** **1**a,b (additional images in Figure S4a, Supporting Information). In addition to the lighter and larger spots of star‐like differentiated cells, ascribable to the underlying astrocytes, two major DRG cell morphologies were observed. A population exhibited spindle‐shaped cell bodies with long, extended processes (Figure 1c), resembling peripheral glial cells such as Schwann and satellite cells.^[^ ^58^ ^]^ The other group displayed rounded cell bodies and bifurcated axons, characteristic of the pseudo‐unipolar DRG neurons^[^ ^2^ ^]^ (Figure 1d and Figure S4b, Supporting Information). The tilted SEM image in Figure 1e (also Figure S4c,d, Supporting Information) reveals DRG neuron‐like cells adhering to the underlying dense mat of nanowires, which provided mechanical support without structural failure despite their thinness. Additionally, several nanowires (indicated by red arrows in Figure 1e) appeared to be incorporated into the cell membrane creating multiple tight contact points cell membrane‐nanowires, which are expected to enhance the ability of effectively capturing cellular electrical activity. The distribution of the soma diameters for the DRG neuron‐like cells (Figure 1f) highlighted a prevalence of small‐sized cells, with a mean diameter of ≈11 µm, which is consistent with the C‐fiber nociceptor type (diameters between 10–30 µm).^[^ ^6^, ^7^ ^]^

#### 2.1.3 Immunofluorescence Analysis

To confirm the nature of the DRG cell population suggested by SEM images, we examined the expression patterns for growth associated protein 43 (GAP‐43), a marker for neuronal growth cones and axonal regeneration^[^ ^59^ ^]^ and glial fibrillary acidic protein (GFAP), a marker for astrocytes, and non‐myelinating immature Schwann cells and satellite cells.^[^ ^60^ ^]^

**Figure 2:**
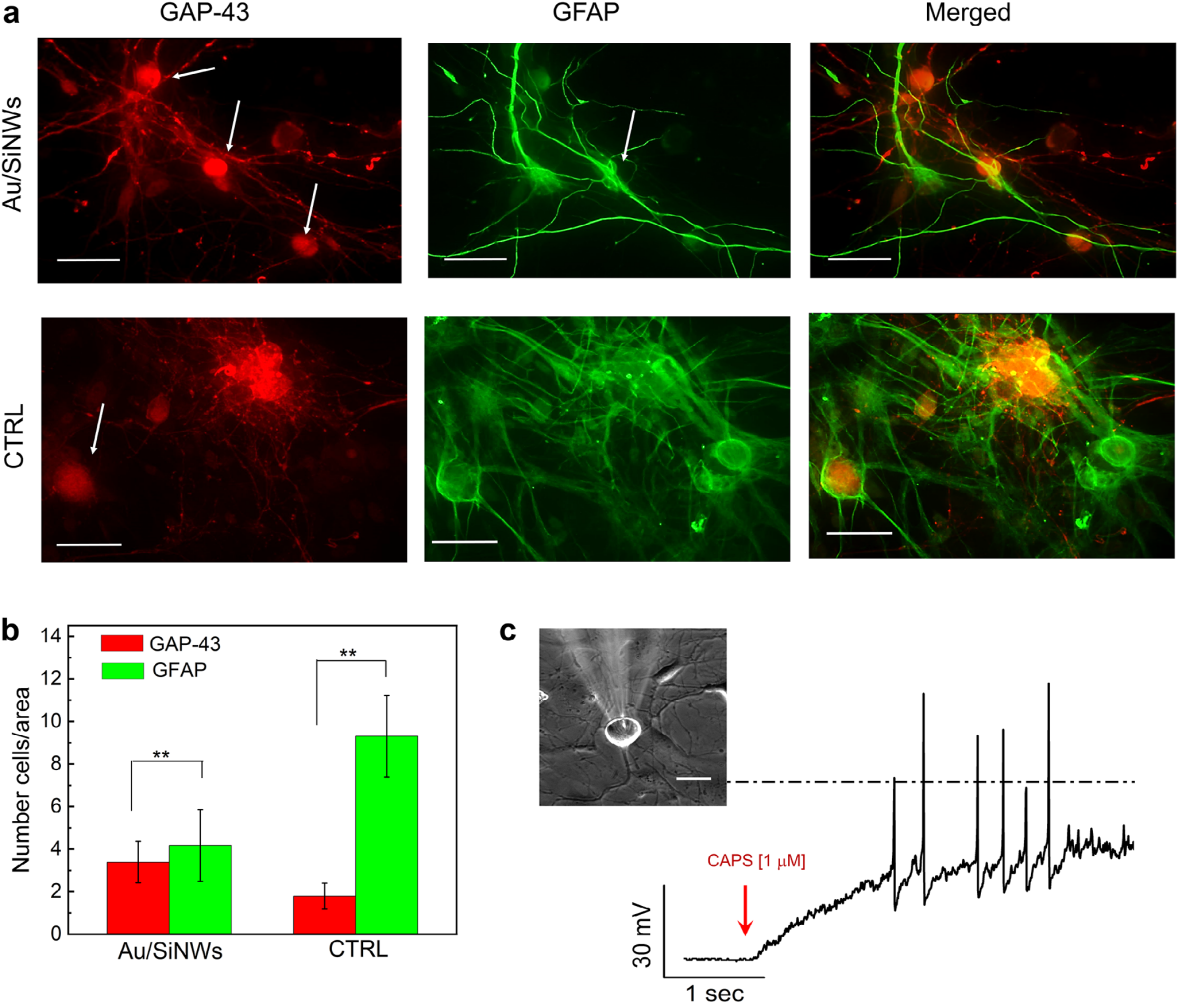
Immunofluorescence and patch‐clamp analysis of the astrocyte/DRG cell culture plated on Au/SiNW substrates: DRG neurons grew on astrocyte feeder layer and differentiated into CAPS sensitive neurons. a) Representative immunofluorescence images of DRG cells plated with astrocytes on Au/SiNW (upper row) and CTRL (lower row) substrates at 5 DIV. Neurons were labeled with GAP‐43 (red staining), and non‐neuronal cells with GFAP (green staining), corresponding to images in the left and central columns, respectively. Merged images are presented in the right column. Scale bar: 50 µm. b) Bar plot reporting the number of cells, counted within the field of view of 40× captured images, positive to GAP‐43 and GFAP on Au/SiNW and CTRL substrates (N = 3 number of experiments). Statistics is One‐way ANOVA. *p < 0.05, **p < 0.01, ***p < 0.001. c) Train of APs from DRG neuron, evoked by CAPS [1µM] addition. The dotted line represents the potential of 0 mV. In the inset, microscope image showing the pipette approaching a DRG neuron in the culture. The DRG neurons selected to patch clamping were those cells with rounded bodies.

Immunofluorescence staining (**Figure** **2**a) showed GAP‐43 (red) and GFAP (green) expression on both Au/SiNW (upper row) and CTRL (lower row) substrates. Furthermore, DRG neurons exhibited characteristic rounded cell bodies (the red stained cells indicated by white arrows) and long axons, while glial cells showed essentially elongated shape cell bodies (the green stained cells indicated by white arrows) with extended projections, aligning with SEM observations (Figure 1a–c). Though star‐like shaped cells, ascribable to astrocytes, were less prominent due to their position beneath the DRG neurons, their presence was confirmed through additional images (Figure S5, Supporting Information) captured from areas of the Au/SiNW mat where the astrocytes were more distinguishable. To further validate the characterization of the cell population, we performed additional immunofluorescence staining using neuronal nuclei (NeuN), a marker specific for neurons’ nuclei, and 4′,6‐diamidino‐2‐phenylindole (DAPI) labeling all cell nuclei. The quantitative analysis obtained with both GAP‐43/GFAP (Figure 2b) and NeuN/DAPI staining (Figure S6, Supporting Information) revealed a relatively higher presence of glial cells in the CTRL compared to the Au/SiNWs. In any case, all immunofluorescence characterizations confirmed the ability of the Au/SiNW mats to support adhesion and growth for both neurons and glial cells.

#### 2.1.4 Electrophysiological Analysis

The functional properties of DRG neurons in the culture were assessed using whole‐cell patch‐clamp. Astrocytes/DRG cells were similarly cultured on both Au/SiNW mats and CTRL. For these experiments, CTRL substrates were poly‐d‐lysine‐coated glass slides to facilitate the visualization of the neurons through a transparent substrate. Bright‐field microscopy (Figure 2c, inset) confirmed precise pipette targeting of DRG neurons. Initially the recordings were acquired in current‐clamp mode following the protocol described in Experimental Section. Comparative analysis of neurons’ passive properties (Table S1 and Note S1, Supporting Information) reveals close alignment between the cultures on both Au/SiNW and CTRL substrates.

Successively, the hypothesized presence of C‐fiber nociceptor phenotype was investigated through stimulation with CAPS, an agonist of the channel TRPV1^[^ ^31^ ^]^ typically eliciting the response of DRG neurons, particularly that of the nociceptors. Accordingly, we measured response to CAPS [1 µM] of DRG neurons (number of the recorded DRG neurons, N = 12) in the cultures on Au/SiNWs (N = 6) and CTRL (N = 6) (more details in Experimental Section). The experiments involved specimens from at least three distinct animals and Au/SiNW substrates from different manufacturing batches; representative AP train is shown in Figure 2c. The electrophysiological properties of DRG neurons with a positive response to CAPS stimulation (reported in Table S2 and commented in Note S1, Supporting Information), including resting membrane potential and other parameters, closely aligned with the values observed in the CTRL group, as well as with those found in the literature for the C‐fiber nociceptor neurons.

Collectively, our results indicate that Au/SiNW mats, with the assistance of astrocytes, effectively supported DRG neuron and glial cell culture by preserving their morphological and bioelectrical properties. Furthermore, the presence of C‐fiber nociceptor neurons within these cultures was confirmed through CAPS stimulation, highlighting the platform's utility as a reliable system for studying chronic pain mechanisms and investigating nociceptor functionality.

### 2.2 Concept and Fabrication of MEA based on SiNW Mats

We designed and developed a multi‐unit MEA based on SiNW mats (NW_MEA). This device included two distinct recording units, designated as U1 and U2. Given the complexity of the cellular population, the main idea of this design was to use one unit to record the activity generated within the population of DRG neurons and glial cells cultured on an astrocyte feeder layer, while the other unit monitored a baseline signal from an astrocyte monoculture under identical environmental conditions by providing a reliable reference for comparison.

**Figure 3:**
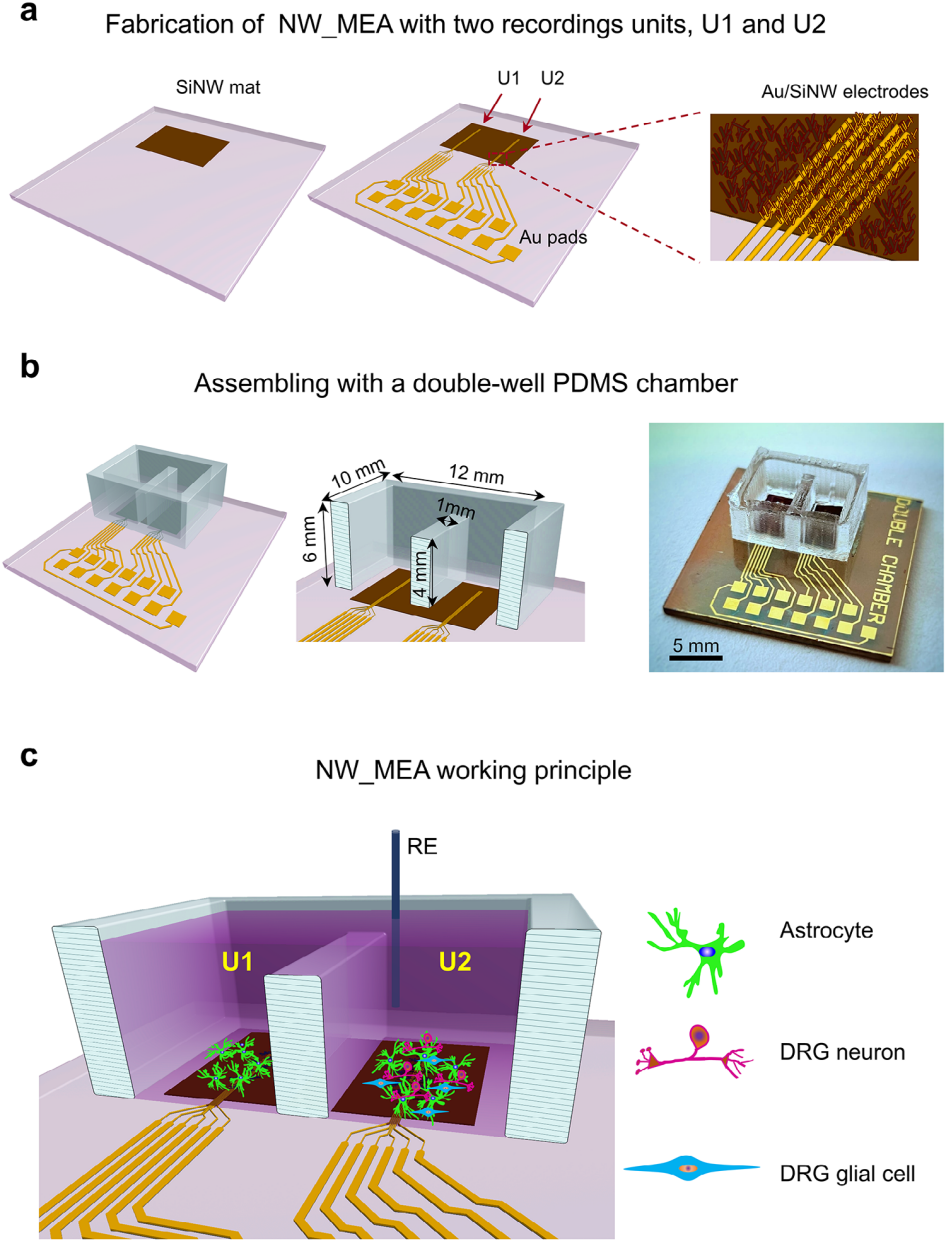
Design of the NW_MEA and its working principle. a) Fabrication steps of NW_MEA with two culturing and recording units: Growth of a SiNW mat on 12 mm x 10 mm selected area of a microscope glass slide by PECVD (left panel); fabrication of two recording units (U1 and U2) and the external Au pads (right panel). Each recording unit is formed by six finger‐shaped electrodes, obtained by evaporating a Ti/Au bilayer onto selected areas of the SiNW mat (yellow stripes in the image of right panel). The electrodes (referred as Au/SiNW electrodes) measured 8 mm in length and 25 µm in width, with a pitch of 50 µm. The units U1 and U2 were spaced 5 mm apart. b) Customized double‐well PDMS chamber placed on top of the two recording units (left and central panels) and photograph of a representative completed device (right panel). c) Schematic illustrating the working conditions with NW_MEA: in U1, astrocytes alone were cultured on and recorded by the electrodes; in U2 a layer of astrocytes supported the growth of DRG cells, including both neurons and glial cells, which were then recorded by the electrodes in U2. A common reference electrode (RE) was shared between both recording units.

**Figure** **3**a illustrates the main fabrication steps (additional details in Experimental Section) of the developed NW_MEA, which builds on a previous version^[^ ^42^ ^]^ with single recording unit, successfully applied to record the electrical activity of neuroendocrine cells. First, SiNWs were grown on a selected area (12 mm x 10 mm) of a microscope glass slide (left panel). Successively, two sets of six finger‐shaped electrodes (25 µm x 8 mm, pitch of 50 µm) and connecting tracks were created by thermal evaporation of a Ti (20 nm)/Au (125 nm) bilayer onto patterned areas of the insulating SiNW mat, as shown in the central and right panels of Figure 3a. Figure S1c,d, Supporting Information, show representative SEM images of a set of these electrodes (referred to as Au/SiNW electrodes) and related close‐up view, respectively.

The planar connecting tracks (Au pads) were used to electrically connect the NW_MEA to the customized low‐noise acquisition system, previously developed^[^ ^42^ ^]^ and shown in Figure S7, Supporting Information.

A customized polydimethylsiloxane (PDMS) double‐well chamber was positioned atop the two recording units, as shown in Figure 3b (additional details in Experimental Section and in Figure S8, Supporting Information). The photograph of a representative assembled device is on the right side of Figure 3b. The PDMS chamber design effectively divided the chamber into two cell culturing and recording compartments. In our experiments, U1 housed the astrocyte monoculture, and U2 housed the culture of DRG neurons and glial cells on the astrocyte layer (Figure 3c). During the recording phase, the culture medium was replaced with a standard external saline (EXT) solution. To connect the two compartments an appropriate volume of solution necessary to overcome the PDMS barrier was added, and both compartments utilized the same reference electrode (RE), as depicted in Figure 3c. This approach enabled simultaneous recordings under similar conditions for both culture types, allowing us to distinguish the contribution of the underlying astrocytes (recorded by both electrode groups in U1 and U2) and DRG cells (solely recorded by the electrode group in U2).

We observe that the design of Au/SiNW electrodes differs significantly from traditional ones, which are typically smaller by few orders of magnitude. Since mechanical interactions between micro/nanostructured surfaces and cells can influence adhesion, proliferation, morphology, and functionality,^[^ ^61^ ^]^ the proposed design offers a simple way to create a MEA with uniform nanotopography, reducing discontinuities between conductive and insulating areas. This uniformity ensures consistent physical features across the MEA surface, fostering a uniform cellular response. Additionally, coupling nanostructured MEAs with multicellular cultures, including neurons and different glial cells, face several challenges associated with typical excessive glial cell proliferation in vitro.^[^ ^62^ ^]^ Undesirable phenomena can occur including encapsulation of electrode nanostructures, inflammatory responses, or scar tissue formation.^[^ ^63^ ^]^ These events may hide bioelectrical activity from most of the neurons in the multicellular culture or prevent their physical contact with electrodes. The larger size of our electrodes mitigates these issues by increasing the contact area with the culture, which in turn enhances the electrode's ability to be electrically coupled with a number of neurons located in close proximity and actively firing. Together, these factors contribute to the platform's ability to effectively record neuronal signals. However, it is important to note that signals recorded from an electrode are the sum of different cell contributions simultaneously active and adhering to it.

### 2.3 Neuronal Signal Recording from Astrocyte/DRG Cell Cultures using NW_MEA under Pharmacological Modulation

#### 2.3.1 Experimental Setup and Baseline Assessment

**Figure 4:**
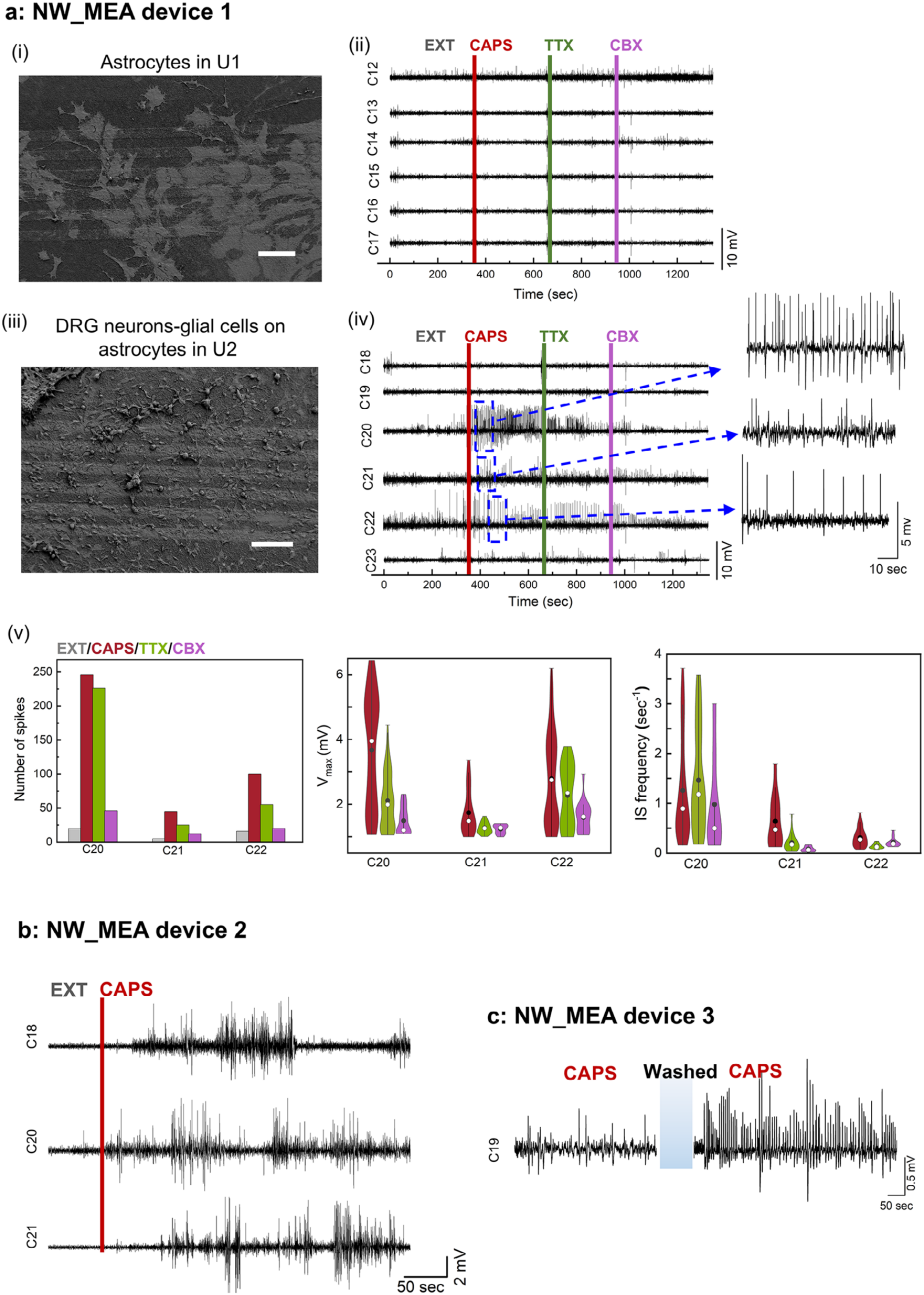
Bioelectrical activity recordings in astrocyte/DRG neuron‐glial cell culture using NW_MEA under pharmacological interrogation. Three independent experiments using NW_MEA devices from different batches, involving N = 3 animals, are illustrated. a) Recordings from NW_MEA device 1: (*i*) SEM image of astrocyte monoculture in U1 compartment (scale bar:100 µm), and (*ii*) raw data of the whole recorded voltage vs. time traces from the six electrodes in U1 (C12‐C17). (*iii*) SEM image of DRG neurons and glial cells plated on astrocytes in U2 compartment (scale bar:100 µm), and (*iv*) raw data of the whole recorded voltage vs. time traces from the six electrodes in U2 (C18‐C23). Periods of activity detected under pharmacological stimulation: EXT solution only, after the addition of CAPS [50 µM], TTX [2 µM] and CBX [50 µM]. In the inset the recording segments of the traces from C20, C21, and C22 electrodes at about 1 min after CAPS addition. (*v*) Quantitative analysis of the spike counts (left panel) during the entire recordings from C20, C21 and C22 electrodes. Only peaks with spike amplitude *V_max_* > 1 mV (four times larger than R.M.S value) were considered. Gray, red, green, and magenta bars represent spike counts in the EXT solution and after the addition of CAPS [50 µm], TTX [2 µm] and CBX [50 µm], respectively. Violin plots in the central and right panels display spike amplitude (*V_max_*) and inter‐spike (IS) frequency distributions, respectively. Black dot and white dot indicate the mean and the median of the distribution, respectively, the vertical lines join the fifth and nineth percentiles. b) Trace segments recordings from three electrodes (C18, C20, C21) in the U2 compartment of NW_MEA_device 2 after addition of CAPS [50 µm] in the EXT solution. c) Representative trace segment from C19 electrode in the U2 of the NW_MEA device 3. The initial weak signaling activity in this culture after CAPS [50 µm] addition was followed by a remarkable activity exhibited after washing, re‐incubation for 20 min, and CAPS administration. A 6 min recording in EXT was performed before CAPS addition to ensure baseline restoration. Only recordings after CAPS addition are shown for comparison. This behavior, specific to this device, may have been due to not fully optimal initial conditions in the extracellular environment, which were restored after the washout. The re‐incubation period likely allowed neuronal stabilization, leading to improved subsequent recordings.

Astrocyte suspensions were plated simultaneously on both U1 and U2 compartments of the NW_MEA and maintained in supplemented astrocyte 10% FBS medium. After 5 DIV, DRG cells were plated into U2, using the same astrocyte medium (as schematic in Figure 3c). Recordings were performed after 5 DIV of DRG cell plating. The coverage of cells on the electrodes was verified by optical microscopy for live cells and SEM analysis of fixed cells before and after the recording, respectively. In U1, a dense layer of astrocytes spread out across the electrodes (**Figure** **4**a(i), Figures S9a,b, Supporting Information). In U2, clusters of astrocytes are identifiable in the underlying light and large spots, and DRG neurons and glial cells spreading over the electrodes (Figure 4a(iii), Figure S9c,d, Supporting Information). Recordings were performed after replacing the culture medium with EXT solution. A 400 µL volume of EXT solution was used to ensure exchange across the PDMS barrier, allowing communication between U1 and U2 (Figure 3c). Signals were recorded from both the astrocyte monoculture in U1 and the astrocyte/DRG neuron‐glial cell culture in U2 during four sequential pharmacological interventions over 25 min. Figure 4a(ii) shows voltage traces from U1 (astrocyte monoculture), and Figure 4a(iv) shows traces from U2 (astrocyte/DRG neuron‐glial culture). The signals recorded in the EXT solution exhibited noise levels, with root mean squared (R.M.S.) values, averaged over six electrodes, of 270 µV in U1 and 280 µV in U2. These values are approximately five times higher than the R.M.S. value observed before cell culture (50 µV; Note S2 and Figure S10, Supporting Information) highlighting the impact of cell adherence on electrode performance. Furthermore, the similar values obtained from electrodes covered with either astrocytes alone or astrocytes and DRG cells suggest that baseline noise primarily originates from the astrocyte layer due to their larger cell size compared to DRG neurons and glial cells.

#### 2.3.2 Recordings under Pharmacological Modulation

Pharmacological administrations were conducted to investigate neuronal activity in the DRG neuron‐glial cell population. Based on the patch‐clamp findings, suggesting the presence of C‐fiber nociceptor phenotypes, we first investigated the effect of CAPS. After 6 min of recording in EXT solution, CAPS [50 µM] was added. CAPS addition to the astrocyte/DRG cell culture elicited several spikes, primarily on channels C20, C21, and C22 (Figure 4a(iv)). These spikes reached amplitudes of several millivolts, significantly larger than those typically recorded in DRG cell cultures by using MEA technology,^[^ ^20^, ^21^, ^22^, ^23^, ^24^, ^25^, ^26^, ^27^ ^]^ and exhibited a quasi‐periodic pattern. This finding highlights the NW_MEA's enhanced sensitivity in detecting neuronal activity. The addition of tetradotoxin (TTX) [2 µM], a neuronal activity inhibitor^[^ ^64^ ^]^ that blocks voltage‐gated sodium channels (Nav), reduced the amplitude and frequency of neuronal spikes but did not completely suppress them. Complete suppression was achieved only after the addition of carbenoxolone (CBX) [50 µM], which inhibits connexin 43 (Cx43), a gap junction protein expressed by both astrocytes and satellite glial cells.^[^ ^65^ ^]^ In contrast, the astrocyte monoculture in U1 (Figure 4a(ii)) showed no discernible changes with respect to the baseline noise level, indicating that the recorded signals in U2 originated from neurons. Control experiments using planar Ti/Au electrodes on glass slides, designed identically to the NW_MEA, recorded unremarkable signals from astrocyte/DRG neuron‐glial cell cultures (Figure S11, Supporting Information), plated using the same protocol. This suggests that the enhanced recording capability of the NW_MEA is due to improved cell‐NW coupling.

Quantitative analysis performed on recordings from C20, C21 and C22 shows consistent trends across all three electrodes (Figure 4a(v)). CAPS addition significantly increased the number of spikes, which showed a weak reduction upon TTX addition and an abrupt reduction after CBX administration (left panel, Figure 4a(v)). A broad distribution of spike amplitudes (V_max_) was observed for each channel after CAPS addition, indicating contributions from multiple neurons (central panel, Figure 4a(v)). The inter‐spike (IS) frequency distribution (right panel, Figure 4a(v)) exhibited a tendency to lower frequencies, with minimal differences following exposure to CAPS and TTX.

The persistence of signals after TTX exposure suggests that DRG neurons in our culture express both TTX‐sensitive and TTX‐insensitive NaV channels. This finding aligns with previous studies^[^ ^66^ ^]^ showing that the DRG neuron population contains multiple types of voltage‐gated NaV channels with distinct pharmacological properties. Thus, it not only highlights the heterogeneity of the neuronal population in our culture but also indicates that our electrodes are capturing the activity from various types of neurons and can distinguish their different behaviors when pharmacologically modulated. On the other hand, the complete signal inhibition after CBX treatment highlights the potential involvement of glial‐neuronal interactions in modulating the activity of the DRG neurons sensitive to CAPS, accordingly with emerging evidence of glial cells' active roles in pain mechanisms.^[^ ^53^, ^54^, ^55^ ^]^ Understanding these interactions is crucial for developing new treatments for various neurological disorders where neuron‐glial interactions are disrupted. While acknowledging the significance of these results, a thorough investigation is necessary that is beyond the scope of the present work.

Successful recording experiments were obtained from several different astrocyte/DRG neuron‐glial cell cultures, involving specimens of distinct animals and devices from different manufacturing batches (details in Experimental Section). Figure 4b,c show representative recordings from the astrocyte/DRG neuron‐glial cell populations on two different devices, NW_MEA device 2 and NW_MEA device 3: cells responded coherently to pharmacological neuronal stimulation although minor discrepancies were observed between different experiments. These discrepancies can be ascribed to the variability in the nanowire‐neuron interfacing efficiency. In addition, DRG neurons are a heterogeneous population, consisting of different neuronal types and subtypes with distinct physiological properties.^[^ ^8^, ^66^ ^]^ This intrinsic variability in cellular behavior can influence the recorded signals, resulting in slight differences in the amplitude of the spikes observed and response to administrated agonist and antagonist drugs.

#### 2.3.3 Spike Shape Analysis and Comparison with Patch‐Clamp Recording

**Figure 5:**
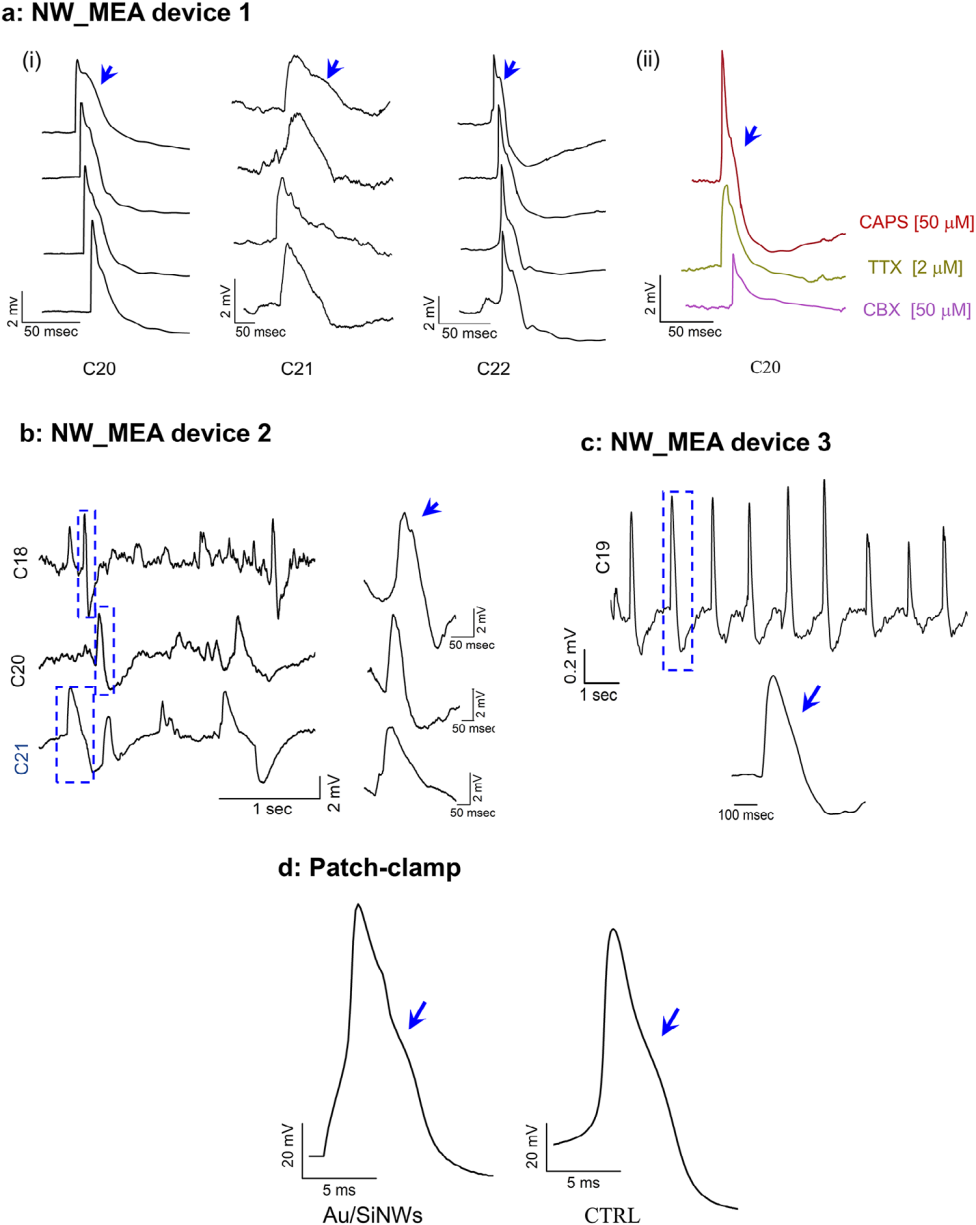
Spike shape analysis and patch clamp comparison. a) Three sets of four representative single spikes randomly extracted from the traces recorded by C20, C21, and C22 of NW_MEA device 1 (shown un Figure 4a(iv)) after CAPS addition (i). Representative individual spikes randomly extracted from C20 after adding CAPS (red line), TTX (blue line), and CBX (magenta line) (ii). The spike shapes in (i) and (ii) were all similar, exhibiting typical AP waveforms. A shoulder occurred in the falling phase of all the traces, as highlighted by the blue arrows. b,c) Close‐up views of selected time segments from C18, C20, and C21 of NW_MEA device 2 (shown in Figure 4b), and from C19 of NW_MEA device 3 (shown in Figure 4c), respectively, after CAPS [50 µm] addition. The enlarged regions represent typical spike shapes that still exhibited features consistent with an AP signal and the occurrence of the shoulder in the falling phase of the waveforms as indicated by blue arrow. d) Representative patch‐clamp AP traces recorded from single CAPS‐responsive DRG neuron within the astrocyte/DRG neuron‐glial cell cultures plated on Au/SiNWs (left panel) and CTRL (right panel). Arrows indicate the presence of a shoulder in the falling phase of the AP traces, typical feature of C‐fiber nociceptor neurons.

In **Figure** **5**a(i), three representative sets of four individual spikes, randomly selected from recordings of C20, C21, and C22 of NW_MEA device 1 after CAPS addition are displayed.

Notably, the waveforms exhibited a profile like AP signal, with a pronounced shoulder in the falling phase as indicated by blue arrows. This peculiar waveform persisted even after the addition of TTX and CBX, as shown in Figure 5a(ii) for C20 electrode, and across all conducted experiments involving distinct cultures and NW_MEA devices (Figure 5b,c).

Interestingly, whole‐patch clamp recordings from CAPS‐sensitive DRG neurons in cell population cultured on both Au/SiNW mats and CTRL substrates exhibited similar waveform profiles. These profiles included the characteristic shoulder in the AP's falling phase (Figure 5d) that is the hallmark of C‐fiber nociceptors'signals, attributed to an inward voltage‐dependent calcium current and TTX‐resistant NaV channel current that partially counteract outward potassium currents during the falling phase.^[^ ^30^ ^]^

These findings strongly suggest that Au/SiNWs enable spontaneous and stable intracellular access, consistent with recent observations on similar nanostructures.^[^ ^42^, ^43^, ^67^ ^]^ This capability allows the NW_MEA to reliably capture key electrophysiological features of C‐fiber nociceptor neurons, a task that remains challenging for conventional MEAs.

## 3 Discussion

Overall, our results demonstrate that the NW_MEAs enable reliable and continuous intracellular recordings of drug‐modulated activity of neurons in DRG cultures. In these cultures, where various sensory neuron types and subtypes coexist,^[^ ^8^ ^]^ our NW_MEA offers the potential for simultaneous observation of bioelectrical behavior from multiple nociceptors, simply by tracking those signals showing the shoulder in the recorded traces. This peculiarity provides the opportunity to observe how drug treatments impact nociceptors over time and how their collective responses evolve. Unlike patch‐clamp, which is limited to single‐cell recordings for short periods and requires prior identification of nociceptive neurons, our platform offers a broader, more efficient approach for studying pain transmission and evaluating drug responses in vitro.

We hypothesize that the intracellular access of NW_MEAs occurs through a mechanism like that previously reported.^[^ ^68^, ^69^ ^]^ cell adhesion to the NWs on the Au/SiNW electrode may induce localized plasma membrane deformation, increasing its permeability and leading to nanopore formation in the lipid bilayer. Consequently, the tips or portions of several NWs establish direct contact with the cytosol, capturing intracellular signals proportional to the membrane potential. Figure 1e, along with Figure S4c,d, Supporting Information, suggests indeed that the tips of longer nanowires are in close contact with the cell membrane and may have penetrated it. This process is strongly influenced by the nanowire geometry and the mechanical properties of the cells.^[^ ^68^, ^69^ ^]^ Thus, natural variations in the length and number of internalized NW tips/portions per cell are expected to influence the resulting impedance at the electrode‐cell interface. In turn, variability in NW accesses into the different cells may contribute to the distribution of spike amplitudes observed at each electrode (Figure 4a(v)), as well as the differences across electrodes (Figures 4a(v),b, and 5a) and between NW_MEA devices   . Moreover, the intracellular coupling through the membrane nanopores rather than the complete membrane rupture characteristic of patch‐clamp techniques together with the different electrical properties of the NW/cell, contributes to the observed amplitude and bandwidth differences of signals recorded by NW_MEA and patch‐clamp techniques.

Despite these intrinsic variations, our NW_MEAs are powerful tools for investigating cellular electrical activity offering remarkable advantages over other reported nanostructured electrodes featuring nanopillars or nanowires. First, intracellular recordings from multiple neurons within the cellular culture is achieved without requiring external triggering (electrical or optical) signals^[^ ^48^, ^49^, ^50^ ^]^ or chemical modifications^[^ ^52^ ^]^ for membrane poration. Even in cases where free‐standing SiNW probes achieve spontaneous entry,^[^ ^38^ ^]^ strict control over the nanowire curvature is required, adding complexity to the fabrication process. Second, the recordings are stable over the recording time (lasting up to 25 min in our experiments) whereas other nanoelectrodes, relying on induced membrane poration, typically allow recordings for only a few tens of seconds. Additionally, intracellular recordings were consistently obtained across different devices and specimens from distinct animals, demonstrating the robustness and high sensitivity of our approach.

Finally, the proposed NW_MEAs can be fabricated using low‐cost, high‐yield, bottom‐up silicon technology at moderate process temperatures (≈350 °C), which are compatible with both glass substrates and flexible polymers such as polyimide.^[^ ^70^, ^71^ ^]^ This allows for direct fabrication of the devices on standard microscope glass slides, enabling real‐time optical observation of cell cultures during biological experiments via integration of the NW_MEA in conventional optical microscope.

Importantly, the fabrication of SiNWs on polyimide films^[^ ^71^ ^]^ enables the development of flexible NW_MEA for recording electrophysiological signals in neural organoids, which represent the new frontiers of in vitro cell models.^[^ ^72^ ^]^ Indeed, flexible electrode arrays are considered among the most promising strategies for interfacing with the soft and complex architecture of organoids^[^ ^73^ ^]^ allowing customized device configurations, such as shell‐like, meshes, folding or wrapping structures for the 3D cell cultures. Our approach uniquely can combine the mechanical adaptability of flexible substrates with the enhanced recording performance of nanostructured electrodes, offering a powerful platform for next‐generation organoid–electronics integration.

Several limitations should be considered in this study. First, the mechanism of intracellular access provided by NWs requires further investigation, particularly by tuning the density and size of the SiNWs. Additionally, the electrochemical characteristics of SiNW‐based electrodes should be modeled as a function of NW density and size to optimize device performance.

Second, the larger size of our electrodes, compared to traditional smaller ones, intrinsically limits single‐cell resolution, as each electrode records the electrical activity of multiple neurons adhering to it. However, the large amplitude of the recorded signals facilitates the recognition and sorting of different neural waveform types, allowing to individuate the activity of different neuronal subtypes.

Third, CAPS quantity needed to stimulate electrical activity in DRG neurons cultured on the NW_MEA was 50 times higher than that required with patch‐clamp technique. This difference could be due to CAPS's hydrophobic nature, which may cause CAPS molecules to be absorbed by the PDMS^[^ ^74^ ^]^ of the double‐well culture chamber of our NW_MEA. Additionally, unlike the patch‐clamp technique which works with single cells, our method involves a population of cells that could require different CAPS content in the EXT solution for neuron firing. Further studies to assess this behavior are planned in the future.

## 4 Conclusions

In this study, we demonstrated the ability to achieve continuous intracellular recordings from DRG neurons within a complex and heterogeneous population of CNS and PNS cells, using a nanostructured MEA based on SiNW mats. The SiNW platform provided several key advantages: i) it supports the culture of both primary rat DRG neurons and glial cells, with the assistance of astrocyte feeder layer, preserving their unique morphological, molecular, and bioelectrical properties; ii) it enables intracellular access to the neurons; and iii) it allows stable recording of APs lasting up to 25 min, capturing key details to identify C‐fiber nociceptor neurons, fundamental targets in the chronic pain research.

This work significantly pushes the nanostructured MEA technology to the next level, paving the way for more detailed in vitro studies of neural sensing, transduction, and communication. Testing the NW_MEA with a complex PNS/CNS cell model not only demonstrates the potential of the device but also provides a holistic approach in studying pain mechanism accordingly the growing focus toward the critical role of PNS glial cells and astrocytes in chronic pain.

Finally, the compartmentalized architecture and flexible fabrication technology of the NW_MEA, along with the SiNW mat’ capacity to interface efficiently with diverse cellular populations, make our approach a powerful tool for broad application potential. Beyond research on CNS and PNS co‐cultures, we foresee its use in studies involving genetically modified electrogenic cells and bioengineered tissues, which are vital models in the rapidly advancing field of synthetic biology.

## 5 Experimental Section

### Fabrication of SiNW Mats and NW_MEA

SiNW mats were produced by plasma enhanced chemical vapor deposition (PECVD)^[^ ^75^, ^76^, ^77^ ^]^ on selected 12 mm × 10 mm area of microscope glass slides. To induce the SiNW growth, a 2 nm thick Au film was selectively evaporated onto the specific area, defined using photolithography and wet etching processes. The growth was performed with SiH_4_ and H_2_ as precursors at a total pressure of 1 Torr and substrate temperature of 350 °C. The flow ratio SiH_4_/(H_2_+SiH_4_) was fixed to 1:10. A 13.6 MHz radio frequency with power fixed at 5 W was used to ignite the plasma. The growth time was fixed at 7 min. A mat of dense, randomly arranged, and oriented SiNWs, approximately long 2–3 µm, with an average diameter at the bottom of about 50–80 nm, were obtained like shown in Figure S1a, Supporting Information.

For the preparation of the MEA, the SiNW mat was covered by a passivating SiO_2_ layer (50 nm thick) deposited by electron cyclotron resonance (ECR)‐PECVD at room temperature, starting from a gas mixture of O_2_, SiH_4_ and He. The insulating film was deposited at a working pressure of 4 × 10^−3^ mbar and at microwave power of 800 W. Successively, a Ti (20 nm)/Au (125 nm) bilayer was evaporated to produce the Au/SiNW electrodes and connecting tracks (Figure 3a), obtained through a lift‐off process by using just acetone. To effectively remove the photoresist from the sample surface, the lift‐off process was performed for 48 h. This time period allowed the acetone to reach and completely remove the photoresist from the less exposed zones of the NW mat. The thin layer of Ti served solely to improve the adhesion between the Au layer and the substrate. The underlying SiO_2_ coating of the SiNWs avoids any short circuit between the created Au/SiNW electrodes. The Au/SiNW electrodes measured 8 mm in length and 25 µm in width, with a pitch of 50 µm. The SEM images in Figure S1c,b, Supporting Information provide the top view of the Au/SiNW electrode array within a single unit.

### Polydimethylsiloxane (PDMS) Double‐Well Culture Chamber

The fabrication of the double‐well chamber involved the design and production of a custom two‐piece mold, illustrated in Figure S8, Supporting Information. The mold was created through 3D printing using an Ultimaker S5, with ABS (acrylonitrile‐butadiene‐styrene) serving as the build material. To enhance the smoothness of the mold surfaces, improve the overall quality and yield of the chamber fabrication process, the mold pieces were subjected to acetone vapors. Prior to pouring the liquid PDMS, the molds underwent thorough cleaning with isopropyl alcohol and deionized water to eliminate any potential residual acetone. PDMS was prepared by mixing silicone elastomer and silicone elastomer curing agent in a 10:1 ratio, both obtained from Sylgard. The solution underwent stirring for 30 min, followed by sonication for another 30 min to eliminate air bubbles, before being poured into the ABS mold. The mold was then placed on a hot plate at 50 °C for a minimum of 4 h and left to rest overnight at room temperature (RT), to guarantee and optimal curing of the PDMS. Subsequently, the PDMS double chamber was carefully extracted from the chamber's edges. Once completed, the chamber is bonded to the glass surface of the device by liquid‐phase PDMS. The last step in the process is a 2‐h drying on a hot plate at 120 °C.

### Rat Cortical Astrocytes and DRG Cells: Culture Preparation, Maintenance, and Plating

Primary cultures of astrocytes were prepared from newborn rat pups (Sprague Dawley) between postnatal days P 0–2 as described in ref.[78] Briefly, neonatal cerebral cortices devoid of meninges were gently triturated, filtered with a 70 µm cell strainer and plated in T25 cell culture flasks containing Dulbecco's Modified Eagle Medium (DMEM) with GlutaMAX and high glucose supplemented with 15% fetal bovine serum (FBS) and penicillin‐streptomycin at 100 U mL^−1^ and 100 µg mL^−1^, respectively. Flasks were maintained in an incubator at 37 °C, 5% of CO_2_, and proper humidity levels for 2 weeks. During this period, we replaced cell medium every 2 days, and flasks were gently shaken when necessary to remove undesired microglial cells. When confluence was reached, astrocytes were dispersed using trypsin‐EDTA 0.25%, and the cell suspension was re‐plated on the NW mats, NW_MEA and CTRL substrates as elsewhere described.^[^ ^46^ ^]^ Cells were plated at a density of 5–7 × 10^3^ per dish and maintained in culture medium containing 10% FBS. After 5 DIV from the re‐plating cells proliferated and grown as a feeder layer, DRG cells were plated while still using the astrocyte medium. Primary cultures of DRG cells were prepared from post‐natal P11‐21 rats according to protocols that we have elsewhere described.^[^ ^79^ ^]^ DRG cells were seeded as follows: an equal amount of the final cell suspension was dropped onto the Au/SiNW mats pre‐coated with astrocytes cells and placed in a 37 °C, 5% CO_2_ incubator. Cells were maintained in DMEM, Gibco supplemented by FBS (10%) in the presence of NGF (50 ng mL^−1^) and avoid cytosinea‐D‐arabinofuranoside (AraC, 1.5 mg mL^−1^) to not reduce the peripheral glial cell expression. Cell cultures were characterized after 3, 5, and 7 DIV by optical and confocal imaging, by scanning electron microscopy (SEM) and patch clamp.

All the experiments were performed according to the Italian law on protection of laboratory animals, with the approval of a bioethical committees of the University of Bologna and of the Ministry of Health (ID 1138, code number 2DBFE.N.3CN, ex‐protocol number 360/2017‐PR) and under the supervision of the veterinary commission for animal care and comfort at the University of Bologna. Every effort was made to minimize the number of animals used and their suffering.

### Cell Viability

The biocompatibility of the cell cultures on both AuSiNW mats and planar Si substrate covered by the same Ti/Au bilayer, used as CTRL, was evaluated at 3 and 7 DIV by performing fluoresceine diacetate assay (FDA). Quantitative analysis was carried out by counting all the live (green) cells using Imagej software, discerning FDA‐positive cells plated on Au/SiNW and CTRL substrates.

### Culture Immunostaining and Confocal Microscopy

The expression levels protein 43 (GAP‐43), glial fibrillary acidic protein (GFAP), and neuronal nuclei (NeuN) in the co‐cultures plated on both CTRL and Au/SiNW substrates were assessed. GAP‐43 is a protein encoded by the gap‐43 gene, characterized as a growth or plasticity protein due to its elevated expression levels in neuronal growth cones during developmental phases and axonal regeneration. On the other hand, GFAP is a well‐established marker for astrocytes, and it is also expressed in non‐myelinating immature Schwann cells and in the satellite cells (PNS).^[^ ^59^ ^]^ NeuN is a marker of mature neurons, predominantly localized in the nucleus of some neurons.

For immunofluorescence staining, cell cultures plated on different the substrates were fixed with paraformaldehyde (4%) in phosphate‐buffered saline (PBS, 0.1 M) for 15 min at RT (20–24 °C). After blocking with bovine serum albumin (BSA, 0.03%) and after permeabilization with Triton X100 (0.01%) in PBS for 30 min at RT. The samples were then incubated overnight at 4 °C with the primary antibodies (Abs) including rabbit anti‐GFAP (1:500, Abcam), mouse anti‐GAP43 (1:2000, Sigma Aldrich); the secondary ABs used for immunofluorescence included Alexa 488‐conjugated donkey anti‐rabbit at and Alexa Fluor 595‐conjugated donkey anti‐mouse (Molecular Probes‐Invitrogen,1:1000 dilution). To identify neurons nuclei, the neuronal nuclear protein (NeuN) staining was performed by using anti mouse NeuN (Abcam, 1:250 dilution) and Alexa 595‐conjugated donkey anti‐mouse (Molecular Probes‐Invitrogen,1:1000 dilution) as secondary Abs. Coverslips were then mounted with Prolong Anti‐Fade, and with 4′,6‐diamidino‐2‐phenylindole (DAPI, Molecular Probes‐Invitrogen) for staining nuclei. The optical images were acquired with a FLUO‐Spin Up Crest Confocal Microscope equipped with a 40 X objective and Photometrics camera, (Crisel Instruments).

### Scanning Electron Microscopy

The morphology of the SiNW and Au/SiNW mats, as well as the NW_MEA device, was verified by a field emission scanning electron microscopy (FESEM) (ZEISS SIGMA 300) at an accelerating voltage of 5 kV. The size of the NWs was determined by combined measurements from the top and cross‐section views.

To observe the immobilized cells onto the substrates and the NW_MEA, they were fixed with 2.5% glutaraldehyde I PBS at 4 °C, for 1 h. Each sample was then rinsed three times in PBS for 5 min before being stained 1 h in 1% Osmium tetra‐Oxide (OsO4) at room temperature; three further rinsing with distilled water were then performed. Then, the samples were sequentially dehydrated in 50%, 75%, 95%, and 99% ethanol. Dried specimens were coated with an evaporated gold (10 nm thickness) before analysis with the FESEM. The observations were performed at an accelerating voltage of 5 kV. Neuron diameters were obtained from 10 plan‐view SEM images using the line tools of the image analysis program ImageJ. The DRG neurons were identified by their rounded bodies. To generate the size distribution, ≈200 diameter measurements were taken, involving three different astrocyte/DRG neuron‐glial cell cultures and specimens from three distinct animals. Histograms were constructed using bin sizes that produced ≈20 bins across the entire distribution. The histogram distributions were then fitted to Gaussian function using Origin data processing software (OriginLab Corp.).

### Current‐Cell Patch Clamp Electrophysiology

Whole‐cell patch‐clamp technique in current‐clamp mode was configured to record APs generated by single DRG neurons seeded with glia cells and astrocytes on both Au/SiNW substrate and glass coverslips coated with poly‐D‐lysine, used as control (CTRL). DRG neurons were voltage‐clamped at a holding potential of −60mV in EXT saline solution. Both CTRL and Au/SiNW substrates were housed within Petri dishes during the measurements. Patch pipettes were prepared from thin‐walled borosilicate glass capillaries to obtain a tip resistance of 2–4 MΩ when filled with the EXT solution. Subsequently, a series of current pulses from 25 to 250 pA amplitude, with incremental steps of 25 pA and a duration of 100 ms, were injected to elicit APs. Neuronal firing occurred in response to threshold current injection, leading to neuron depolarization. Membrane currents were amplified (MultiClamp 700B), filtered at 2 kHz and acquired at a sample rate of 5 kHz by Axopatch 200B amplifier in voltage‐clamp mode. Responses were amplified, low‐pass filtered at 1 kHz, digitized at 20 kHz, stored, and analyzed with pCLAMP 10. The calculation of APs and the electrophysiological properties was performed as previously described^[^ ^80^ ^]^ along with a comparative analysis of the key electrical passive features exhibited by DRG neurons on both the CTRL and Au/SiNW substrates (Table S1, Supporting Information). Recordings of cells not expressing AP were not considered for the study. The maximal number of firings was calculated by counting the number of overshooting AP peaks in response to a 100 ms pulse of current injection. Experiments were carried out at RT. In addition, to test the sensitivity of single DRG neurons to CAPS we performed whole‐cell patch clamp in current‐clamp mode from exposed cells to CAPS [1 µm]. CAPS was continuously applied to the cells using a local gravity perfusion system for 5 about sec in the EXT solution. Wash out (WO) with EXT solution was applied in each set of experiments to restore the initial holding potential (mV), after the depolarization induced by CAPS responsiveness of DRG.

### Data Acquisition System for Recording with NW_MEA

We used a customized recording platform, previously developed,^[^ ^42^ ^]^ connected to a digital acquisition (DAQ) system. In Figure S7, Supporting Information, detailed description of the complete platform. Briefly, it consisted of an aluminum recording box that housed the NW _MEA with the culture chamber and mounted an electronic interface board connected to the DAQ system. The DAQ was composed by a commercial mother + child development board based on Intan Technologies neural chip RHD2216, in turn connected a PC software for data storage and visualization (Intan RHX). The system can perform 16‐bit acquisition on 16 channels at 30k Sample/s per channel. Moreover, digital band pass filter can be set with lower cut‐off frequencies in the range 0.1 ÷ 500 Hz and upper cut‐off frequencies in the range 100 Hz ÷ 20 kHz to limit noise interferences and for offset removal. The chip was designed for intracortical or ECoG recording and had a typical low input‐referred noise of 2.4 µVrms. The board was provided with a co‐axial connector to enable the use of an external reference electrode with the minimum injection of noise in the system. An external platinum wire was bonded to the core of the coaxial electrode and used as reference during the measurements.

### Neuronal Recording with NW_MEA under Pharmacological Drug Application

Astrocyte suspensions were seeded simultaneously on both U1 and U2 of the NW_MEA and maintained supplemented DMEM 10%. After 5 DIV, DRG were plated only into U2, by maintaining the astrocyte monoculture in U1. Recordings were conducted replacing the culture medium with standard EXT solution after 5 DIV of DRG cells plating. The NW_MEAs were placed in a closed aluminum box slot, to limit the noise interference, and connected with the recording board. A platinum reference electrode was inserted in the solution through an *ad‐hoc* hole in the closed box. Hence, the correct alignment between the electronic board and the sample contacts was performed measuring the electric resistance between two pins shorted on purpose on the sample, while the electrical reliability of the contacts was verified through a preliminary impedance analysis at 1 kHz. A heating plate was used to maintain the temperature of the box at 37 °C during the experiment. Finally, the acquisition software was started selecting the following parameters: amplifier bandwidth of 1–7500 Hz, sampling rate = 30k Sample/s. A 50 Hz notch‐filter was adopted to further reduce the interference of the AC cycling. First, 6 min of baseline activity in EXT solution were recorded, followed by 5 min recordings of pharmacological tests: i) CAPS [50 µM]; ii) TTX [2 µM]; iii) CBX [50 µM], added both to U1 and U2. WO with EXT solution was applied each condition.

### Solutions and Chemicals

All salts and chemicals employed for the investigations were of the highest purity grade (SIGMA ALDRICH). For electrophysiological patch‐clamp and electrical recording experiments the standard bath saline, EXT solution, was (millimolar): 140 NaCl, 4 KCl, 2 MgCl_2_, 2 CaCl_2_, 10 HEPES, 5 glucose, pH 7.4 with NaOH and osmolarity adjusted to ≈315 mOsm with mannitol. The intracellular (pipette) solution was composed of (millimolar): 144 KCl, 2 MgCl_2_, 5 EGTA, 10 HEPES, pH 7.2 with KOH and osmolarity ≈295 mOsm.

### Statistical Analysis

*Statistical Analysis—Patch‐Clamp Experiments*: Data are reported as the mean average ± standard error (S.E.) of the number of patched and recorded cells (N). Experiments were performed multiple times (n = 7) on different primary cultures, plated on both fresh CTRL and Au/SiNW substrates. The analyses and statistics of electrophysiological membrane properties were performed with Origin Micro Cal Ver 6.0. Data were compared by one‐way ANOVA with Bonferroni post‐test or student's *t*‐test. * *p* < 0.05, **p < 0.01, ***p < 0.001 independent *t*‐test

*Statistical Analysis—NW_MEA recordings*: Recordings were performed from five astrocyte/DRG neuron‐glial cell cultures, each comprising specimens from three distinct animals and utilizing five devices from different fabrication batches. Data were acquired in binary format (*.rhd) to enhance compression efficiency. Following conversion to ASCII format based on the data structure, a specific analysis protocol was applied to the temporal plots. Initially, a smoothing algorithm was employed using a 50‐point window (1.6 ms window @ 30k Samples/s) applied over the mean value. The chosen window width aimed to minimize the impact of spurious signals on the signal track without significantly altering its amplitude. A peak sorting algorithm was implemented with voltage thresholds set at and 1 mV, and a minimum time frame of 10 ms between two distinct peaks. This strategy effectively reduced the detection of artifact peaks without compromising the retention of meaningful data.

To enhance data accuracy, all peaks identified as artifacts by the peak sorting algorithm were excluded. The resulting data were then utilized to assess event distribution in terms of both frequency and amplitude.

## Conflict of Interest

The authors declare no conflict of interest.

## Author Contributions

I.L., F.M., and G.C. contributed equally. A.C., E.S., V.B., L.M. conceived and designed the experiments. I.L. fabricated the NW_MEA. F. M. designed the NW_MEA and developed the acquisition board. E. P. designed and developed the PDMS double chamber. R.F., G. C. and E.S. prepared the cellular cultures, performed immunofluorescence characterization, viability test and analyzed the related data. A.K., R.F. and C.L. prepared and maintained the astrocytes cell culture. E.S., F.F. and M.C. performed patch‐clamp experiments and analysis. E.S., F.F. and M.C. performed patch‐clamp experiments. E.S., F.F. and V.B. analyze the patch‐clamp data. A. C. performed SEM imaging. I.L., F. M., E.S., G.C., L. M., and A. C. performed electrical recordings with NW_MEA. F.M. and A.C. performed the analysis of the recordings obtained with MEA. A.C. led all aspects of the project and wrote the manuscript. All authors discussed the results and contributed to the manuscript writing and reviewing.

## Supporting information



Supporting Information

## Data Availability

The data that support the findings of this study are available from the corresponding author upon reasonable request.
