# Supplementary material for: Silicon Nanowire Mats Enable Advanced Bioelectrical Recordings in Primary DRG Cell Cultures
Source: Adv Healthc Mater. 2025 May 24;14(17):2500379. doi: 10.1002/adhm.202500379 (PMC12232127; doi:10.1002/adhm.202500379)
Supplement: Supplementary file 1 — Supporting Information [file ADHM-14-0-s001.docx]

**Silicon Nanowire Mats Enable Advanced Bioelectrical Recordings in Primary DRG Cell Cultures**

*Ivano Lucarini^1,§^, Francesco Maita^1,§^, Giorgia Conte^2,§^, Emanuela Saracino^2,^* , Francesco Formaggio^3^, Elena Palmieri^1^, Roberta Fabbri^2^, Aikaterini Konstantoulaki^2^, Chiara Lazzarini^2^, Marco Caprini^3^, Valentina Benfenati^2^*, Luca Maiolo^1^*, Annalisa Convertino^1^**

Supporting information

Table of Contents

Figure S1: Key features of the various SiNW-based mats involved in the study………………………….……2

Figure S2: Immunofluorescence and morphological analysis of DRG cells plated alone on Au/SiNWs..……3

Figure S3: Viability analysis of DRG cells plated on Au/SiNWs with the assistance of astrocytes…..….…...4

Figure S4: Morphological characterization of the DRG cells cultured on Au/SiNWs with the assistance of astrocytes……………………………………………………….………………………………….....………...5

Figure S5: Star-like shaped glial cells in astrocyte/DRG cell culture on Au/SiNWs………….……..….…….6

Figure S6: Confocal characterization of astrocyte/DRG cell culture on Au/SiNWs, stained using NeuN/DAPI ………….………………………………………………………………………………………........................6

Note S1: Current-cell patch-clamp characterization of the DRG neurons within the astrocyte/DRG cell culture…………………………………………………………………………………………………………..7

Table S1: Electrophysiological properties of DRG neurons within the astrocyte/DRG cell culture………...…8

Table S2**:** Electrophysiological properties of CAPS positive DRG neurons within the astrocyte/DRG cell culture………………………………………………………………………………………………………….8

Figure S7: Customized acquisition system………..……………..……………………………………..……...9

Figure S8: Manufacturing process of the double-well PDMS chamber……………………………………….10

Figure S9: Astrocyte monoculture and astrocyte/DRG cell culture morphology on NW_MEA…………..…11

Note S2: Comparison of R.M.S. noise amplitude in NW_MEA with and without cells……………….…….12

Figure S10: NW_MEA recordings without cells………………………………………….………………….12

Figure S11: Bioelectrical activity recordings in astrocyte/DRG neuron-glial cell cultures using a planar MEA under pharmacological interrogation with CAPS……………………………………………………………..13

References…………………………………………………….…………..……………………...…………...14

*
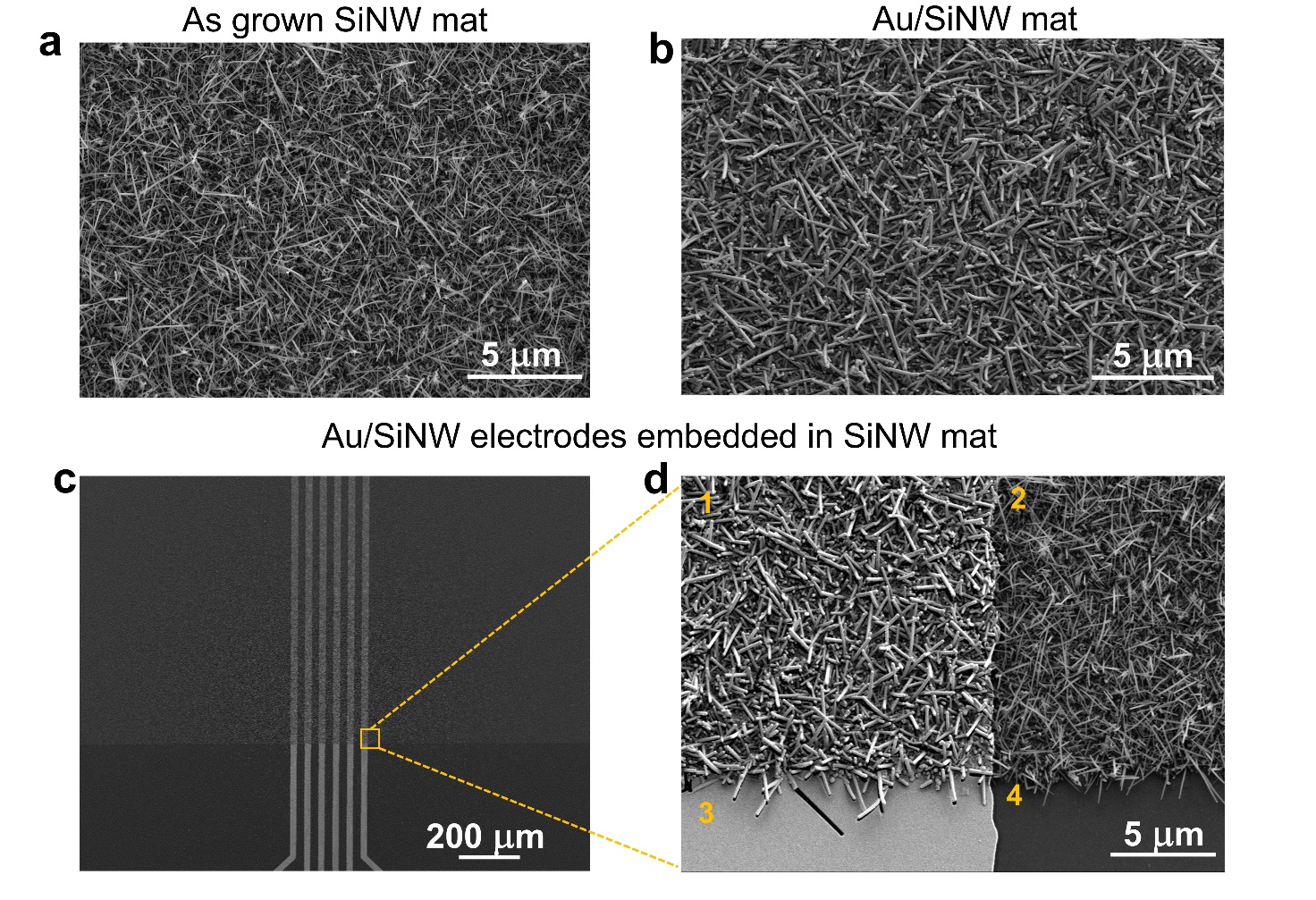
*

**Figure S1**. **Key features of the various SiNW-based mats involved in the study. a** SEM image depicting as grown SiNWs that formed a dense mat of NWs characterized by a length and a diameter at the bottom in the range of 2-3 μm and 50-80 nm, respectively. **b,** SEM image of SiNWs after evaporating Ti(20 nm)/Au(125 nm) bilayer, denoted as Au/SiNW mat. The Au/SiNWs exhibited a more cylindrical shape and an increased average radial size, measuring about 120–180 nm at the bottom. No significant variations in the length were observed. **c**, Representative SEM image of the six Au/SiNW electrodes (light stripes) of the NW_ MEA embedded in the insulating SiNW mat (dark areas). **d**, Close-up view highlighting the interface between Au/SiNWs (area n. 1) and insulating SiNWs (area n.2). The planar Au contacting track (area 3) and the glass substrate (area n.4) are also visible.


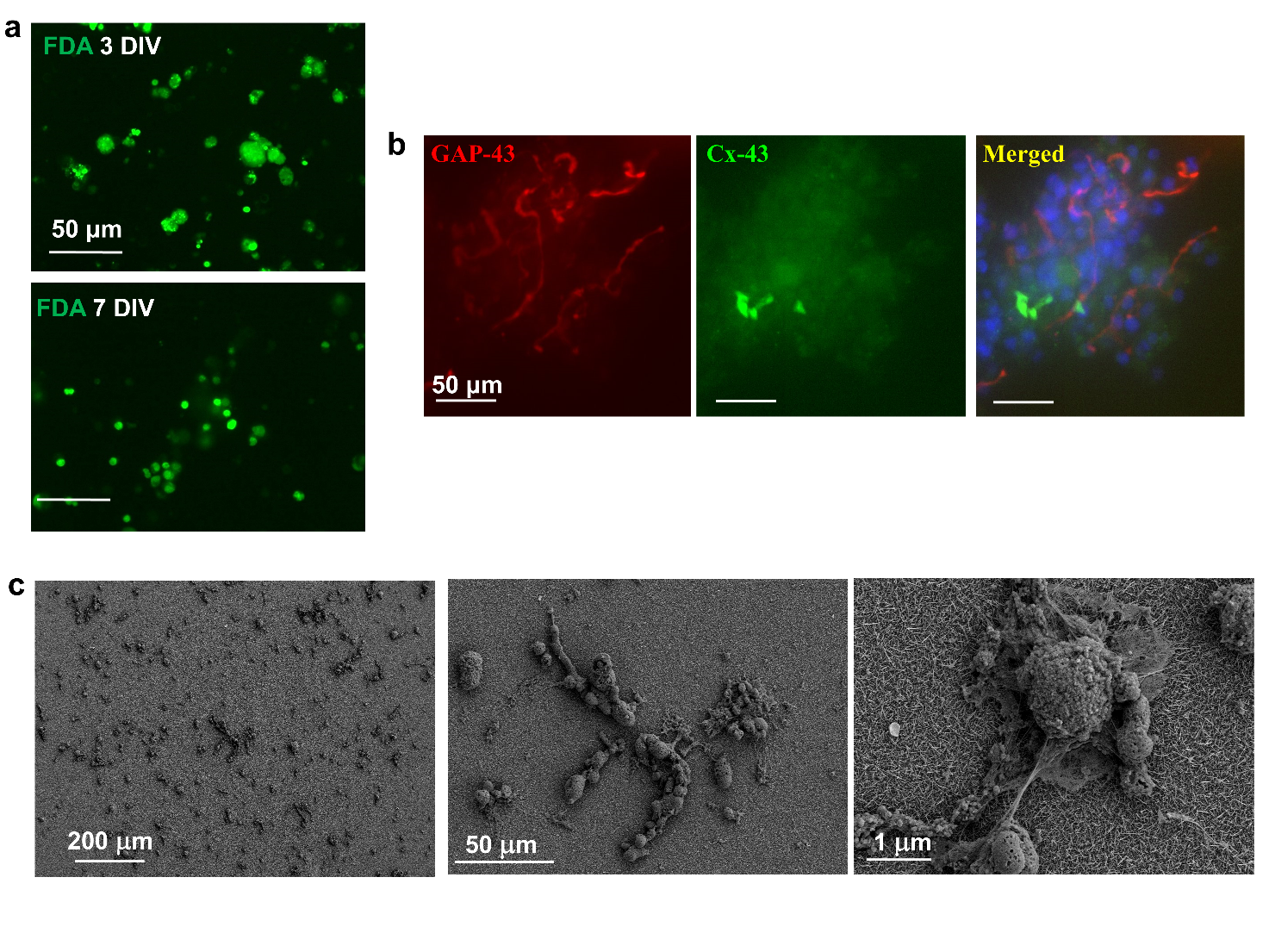


**Figure S2:** **Immunofluorescence and morphological analysis of DRG cells plated alone on Au/SiNWs.** **a,** Fluorescein diacetate **(**FDA) staining images showing DRG cells at 3 days in vitro (DIV) and 7 DIV, with sparse, rounded cell bodies observed on the substrate at both time points. **b,** Low magnification (20X) immunofluorescence images revealing a few positive cells for Growth-Associated Protein-43 (GAP-43), a marker of neural outgrowth and regeneration, and for Connexin-43 (Cx-43), a specific marker for DRG glial cells. **c**, SEM images at different magnifications, showing the formation of only small clusters of cells with spherical body and some filaments departing from these spherical cell bodies suggesting the formation of neurites.


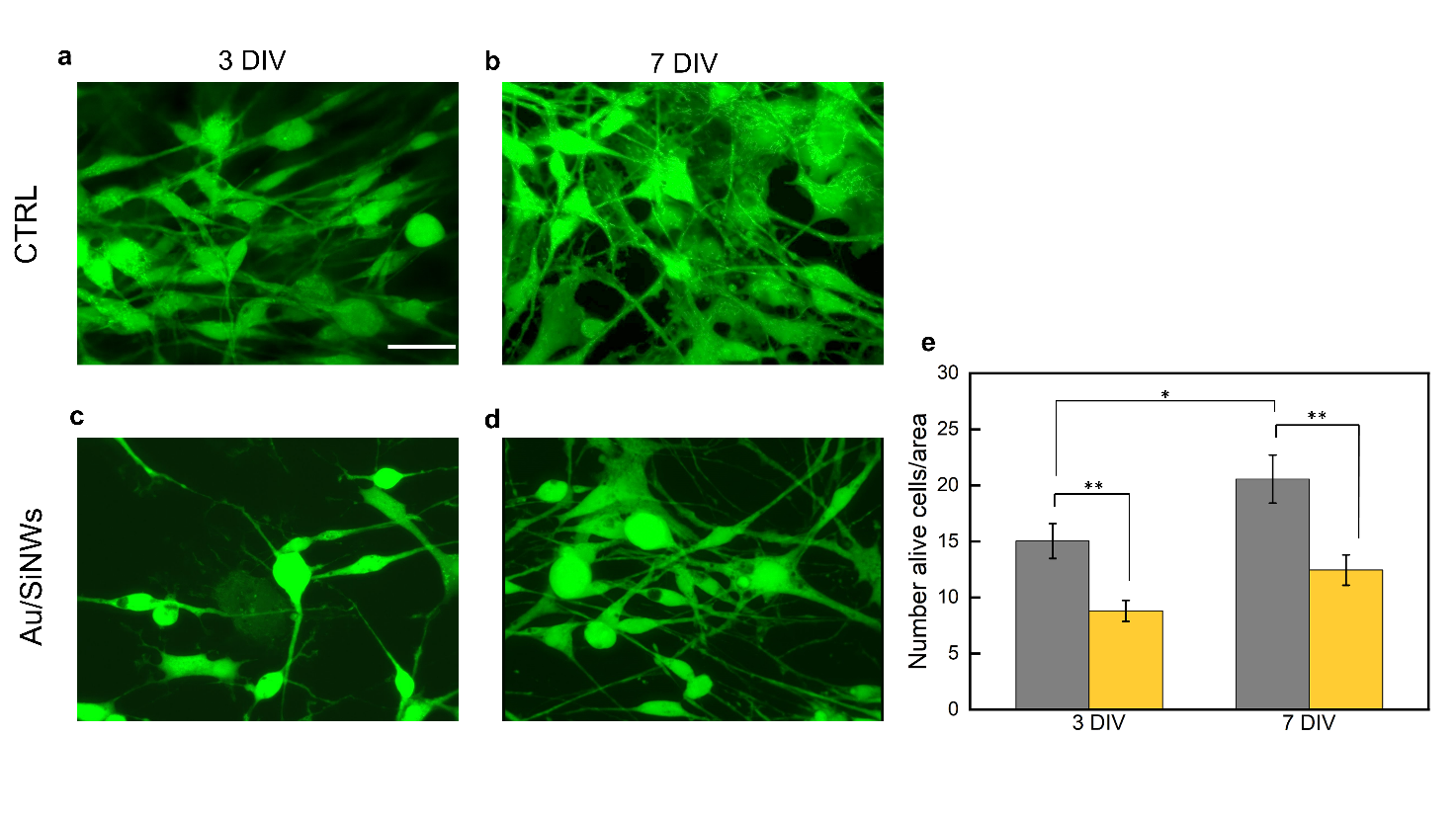


**Figure S3:** **Viability analysis of DRG cells plated on Au/SiNWs with the assistance of astrocytes**. Single-plane 40X confocal images of FDA stained astrocytes and DRG cells plated on CTRL (Au/Si planar, upper row) and Au/SiNW (lower row) substrates at 3 DIV (**a**, **c**) and at 7 DIV (**b**, **d**). Scale bar 50 μm. The living cells were marked in green. **e**, Bar plot reports the number of live cells counted within the field of view of the images from cell culture preparations grown on CTRL (gray bars), and Au/SiNWs (yellow bars), respectively at 3 and 7 DIV. Each set of experiments was repeated in triplicate. Statics is One-way ANOVA. *p<0.05, **p<0.01, ***p<0.001.

 
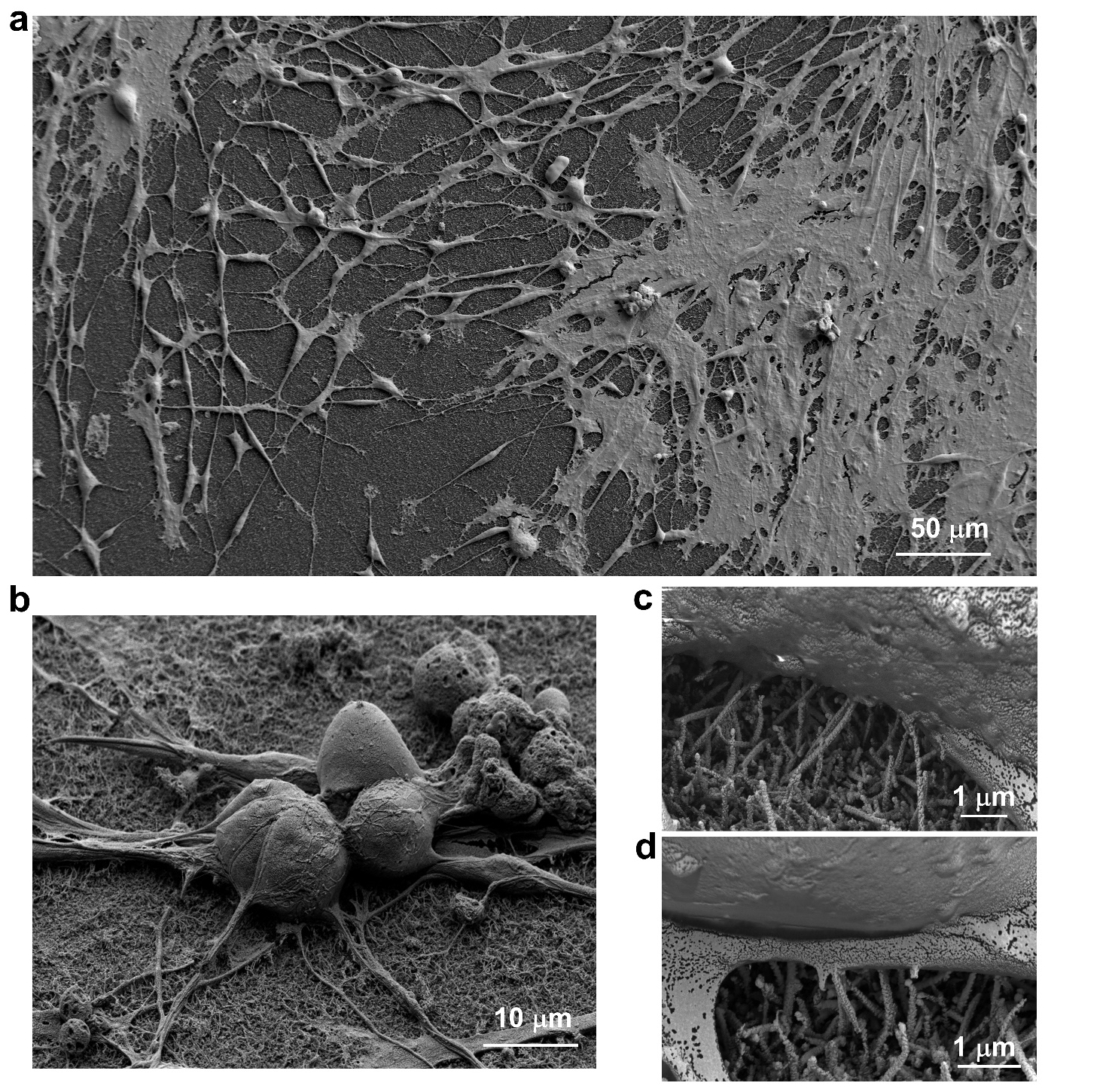


**Figure S4**: **Morphological characterization of the DRG cells cultured on Au/SiNWs with the assistance of astrocytes.** **a,** SEM images showing the DRG cells cultured above astrocytes’ feeder layer, previously plated on Au/SiNWs. Large spots with star-like shape, typical of astrocytes [1], were underneath dense and heterogeneous population of cells, among which cells with a rounded body typical of DRG neurons [2] were visible. The image **b** shows a group of DRG neuron-like cells. **c** and **d**, high magnification images taken at the tilted angle of 30° showing the nanowires below the cell body.


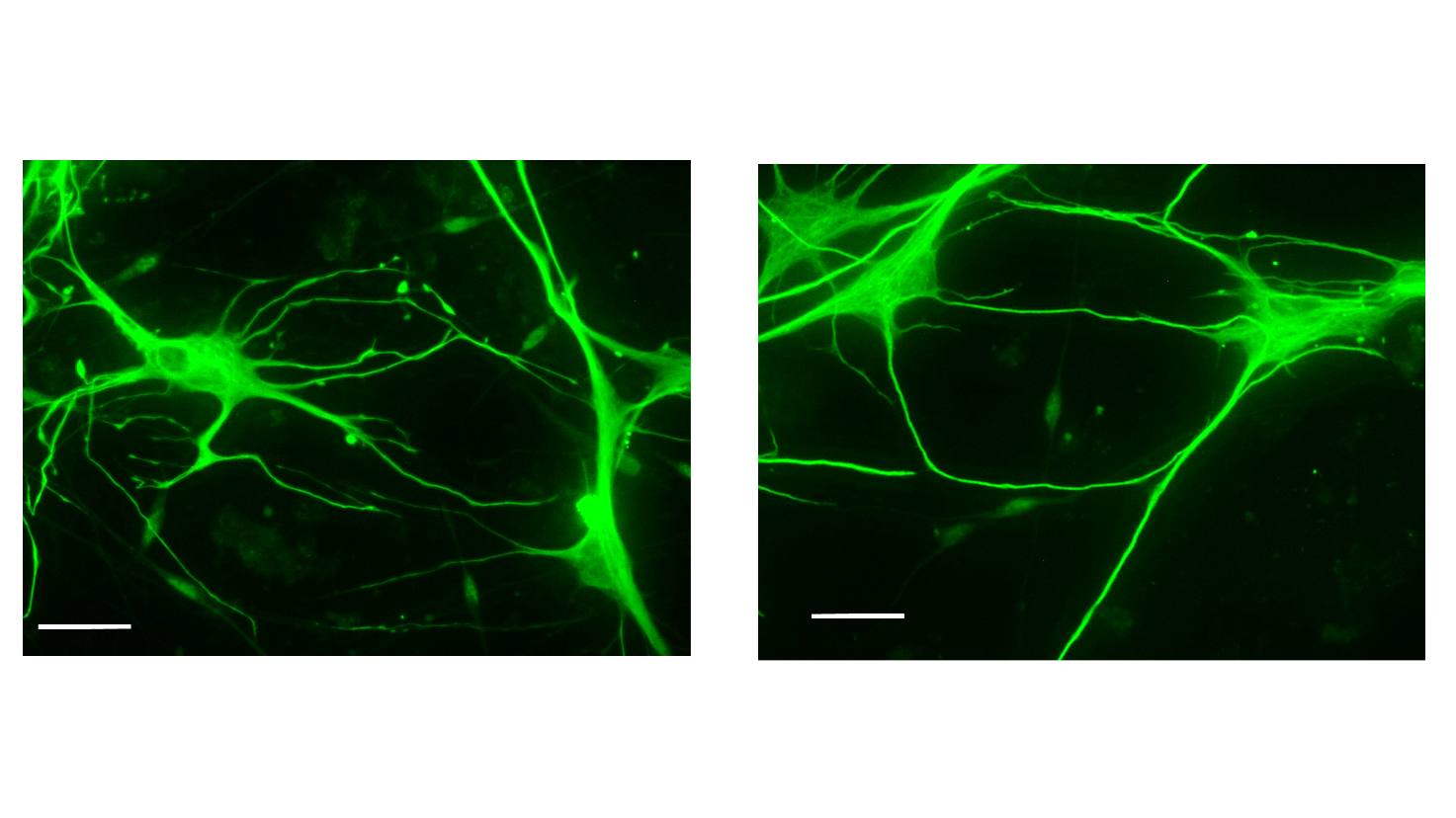


**Figure S5: Star-like shaped glial cells in the astrocyte/DRG cell culture on Au/SiNWs.** GFAP positive cells exhibiting star-like shape with extended processes in the astrocyte/DRG cell culture on Au/SiNWs. Scale bar: 50μm.


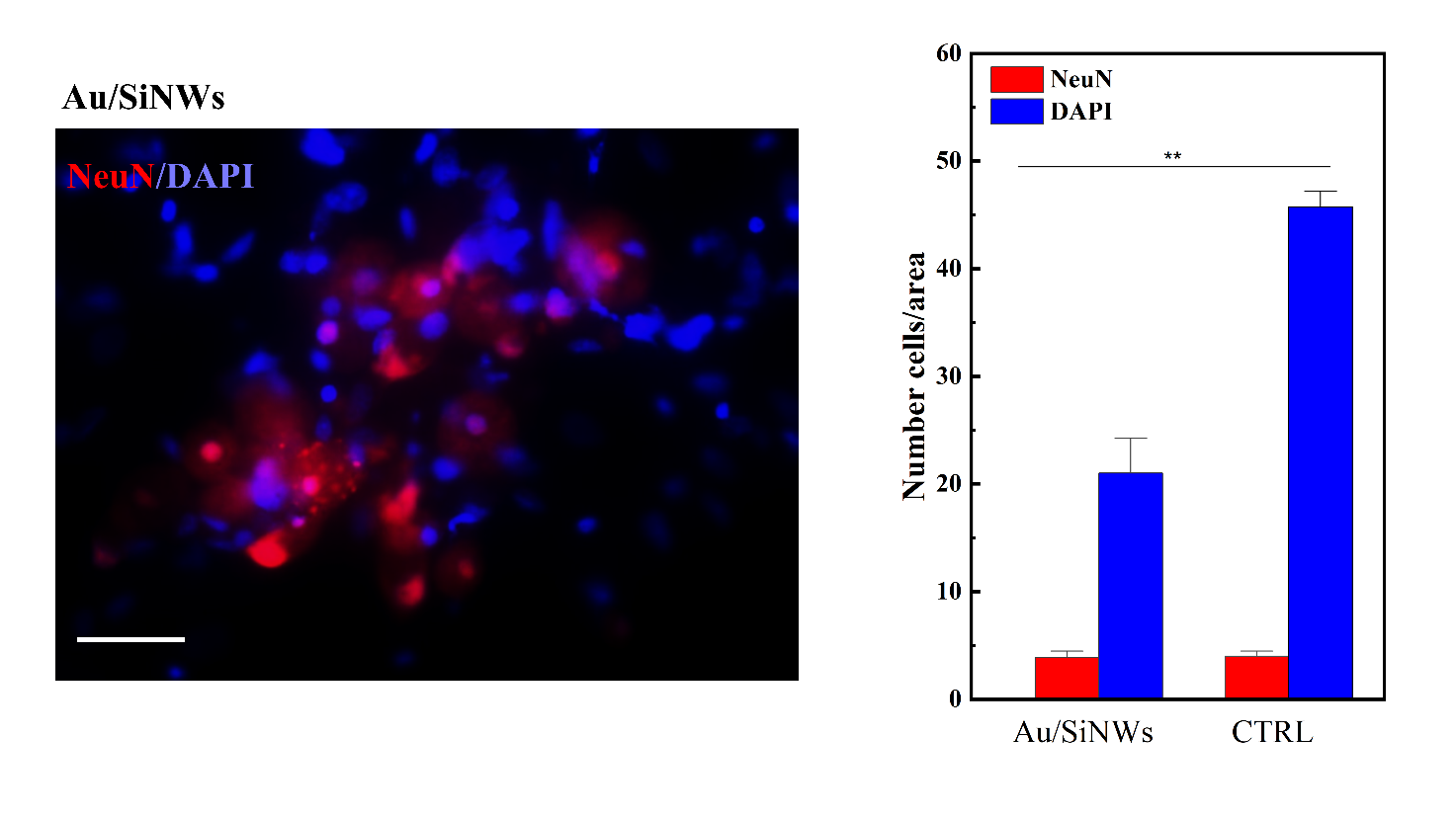


**Figure S6: Confocal characterization of astrocyte/DRG cell culture on Au/SiNWs, stained using NeuN/DAPI. a,** Single-plane confocal image acquired at 5 DIV from DRG cells cultured above astrocytes’ feeder layer, previously plated on Au/SiNWs. 4',6-diamidino-2-phenylindole (DAPI) marks in blue all the nuclei of the co-culture (glia and neurons), while in red neuronal nuclear antigen (NeuN) positive neurons are shown. Bar scale 50 μm**. b,** Bar plot shows the number of DAPI positive cells (blue bars, glia and neurons) and the number of NeuN positive neurons (red bars) counted within the field of view of 20X captured images for both Au/SiNWs and CTRL. Each set of experiments was repeated in triplicate. Statics is One-way ANOVA. *p<0.05, **p<0.01, ***p<0.001.

**Note S1: Current-cell patch-clamp characterization of the DRG neurons within the astrocyte/DRG cell culture**

Whole-cell patch-clamp experiments were performed in current-clamp mode after 5 DIV plating DRG neurons on a layer of astrocytes that had been seeded 5 DIV earlier, on both glass coverslips coated with poly-D-lysine (CTRL) and Au/SiNW substrates, as detailed in Experimental Section. We generated an activation protocol for recording DRG neurons’ APs from a holding potential of −60 mV to -120 mV with 20 mV increment each 100 ms.

Different parameters of APs were considered to compare the excitability of DRG neurons in the CTRL and Au/SiNWs. We analyzed the following AP and after hyperpolarization (AHP) parameters: membrane capacitance (C_p_), resting membrane potential (V_rest_), AP threshold current (I_th_) defined as the minimum intensity of injected current to trigger neuron firing, AP voltage threshold (V_th_) ) that is a membrane potential at an inflection point preceding an AP upstroke. In addition, we measured the AP peak i.e. the overshoot level, the rise time (RT, defined as time from V_rest_ to AP peak), AP duration (APD) measured at the level of V_th_, number (n) of spikes, and AHP amplitude and duration. Table S1 and Table S2 show passive properties of DRG neurons recorded both on CTRL and Au/SiNW substrates. Specifically, Table S1 reports data collected on DRG neurons without discriminating between the different DRG phenotypes and their response to CAPS, while data on Table S2 are strictly related to passive properties calculated in CAPS [1μm] positive DRG neurons. The experiments were performed under same whole-cell patch clamp conditions.

We observe that the V_rest_ mean value, in Table S1 for the DRG neurons plated on both the substrates, lies within the typical range of -60 to -80 mV reported in the literature for DRG neurons [3]. Variations in this range values can occur, reflecting differences in the DRG neuron subtypes, specific conditions under which measurements are performed, and different physiological parameters such as ion concentration gradients, membrane permeability [4]. Specifically, the V_rest_ of CAPS sensitive C-fiber neurons, reported in literature ranges between -50 and -60 mV, reflecting specific ionic properties and channel distributions [5-8]. This observation is in line with our data reported in Table S2.

| **Table S2:** Average of passive electrophysiological properties of CAPS [1 μm] positive DRG neurons within the astrocyte/DRG cell culture on CTRL and Au/SiNWs. N= number of recorded cells. Statics is One-way ANOVA. *p<0.05. | | | | | | | | | | |
| --- | --- | --- | --- | --- | --- | --- | --- | --- | --- | --- |
|  | **Cp**  **(pF)** | **V _rest_**  **(mV)** | **I_th_**  **(pA)** | **V_th_**  **(mV)** | **n**  **spikes** | **AP Peak**  **(mV)** | **RT**  **(ms)** | **APD**  **(ms)** | **AHP amplitude**  **(mV)** | **AHP duration**  **(ms)** |
| **CTRL**  **N=6** | **43±8** | **-53±2** | **46±9** | **-19±3** | **3±0.5** | **59±3** | **3±0.5** | **79±4** | **14±2** | **120±9** |
| **Au/SiNWs**  **N=6** | **32±3** | **-56±2** | **58±6** | **-24±4** | **8±1**  ***** | **45±9** | **3±0.4** | **66±6** | **13±2** | **126±8** |

| **Table S1:** Average of passive electrophysiological properties of DRG neurons within the astrocyte/DRG cell culture on CTRL and Au/SiNWs. N=number of recorded cells. Statics is One-way ANOVA. *p<0.05, **p<0.01 | | | | | | | | | | |
| --- | --- | --- | --- | --- | --- | --- | --- | --- | --- | --- |
|  | **Cp**  **(pF)** | **V _rest_**  **(mV)** | **I_th_**  **(pA)** | **V_th_**  **(mV)** | **n**  **spikes** | **AP Peak**  **(mV)** | **RT**  **(ms)** | **APD**  **(ms)** | **AHP amplitude**  **(mV)** | **AHP duration**  **(ms)** |
| **CTRL**  **N=7** | **62±1** | **-81±2** | **87±7** | **-27±3** | **2±0.2** | **27±5** | **5±0,6** | **53±6** | **18±2** | **137±4** |
| **Au/SiNWs**  **N=6** | **48±7** | **-66±3**  ****** | **97±9** | **-22±2** | **3±0.8** | **34±2** | **5±1** | **55±7** | **14±2** | **146±7** |

All the other properties of DRG neurons on Au/SiNWs closely aligned with the values observed in the CTRL group, as well as those reported in the literature [3]. Collectively these findings suggest Au/SiNWs, with the support of astrocytes, to preserve the bioelectrical characteristics of the DRG neurons, including those of C-fibers nociceptor phenotype.


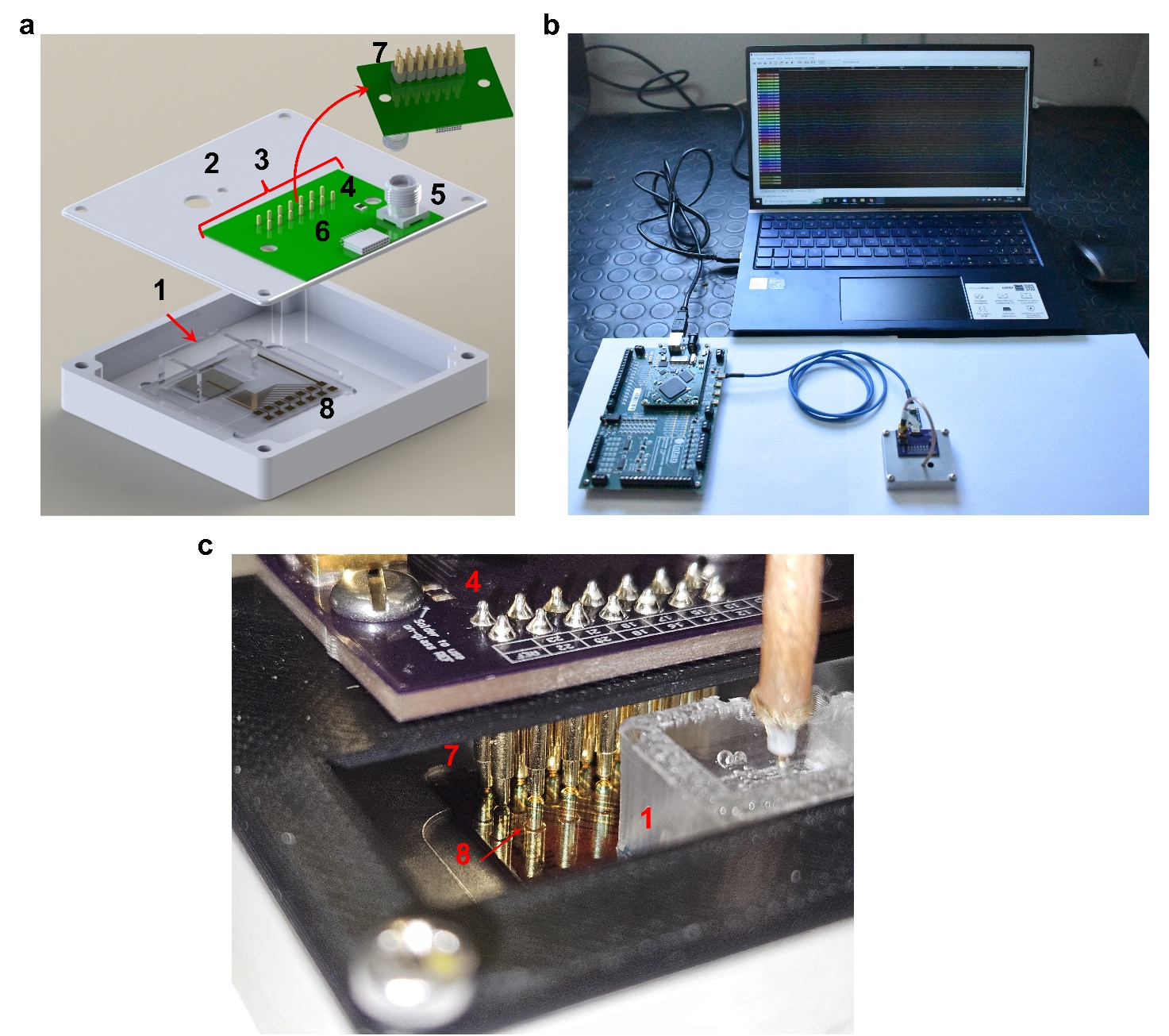


**Figure S7:** **Customized acquisition system. a**, Schematic representation of the customized acquisition system consisting of an aluminum box with a stage for the accommodation of the NW_MEA with the PDMS chamber inside (1). At the top of the box, there was a dedicated hole for the insertion of a pipette to add pharmacological drugs into the PDMS chamber (2). The platform also included the electronic interface board (3), featuring a 2x6 matrix (4) of spring contacts. The inset shows the back of the 2x6 matrix (7). Additionally, the platform incorporated the reference connector (5) and the Intan RHD2216 connector (6). **b**, Photograph of the complete system connected to the Intan RHD2216 board, which was in turn connected to the computer. **c,** Photograph taken inside the acquisition system, visualizing the matrix of spring contacts (7) interfacing with the Au pads of the NW_ MEA (8). The PDMS chamber (1) and the top side of the spring contacts matrix (4) are also visible.


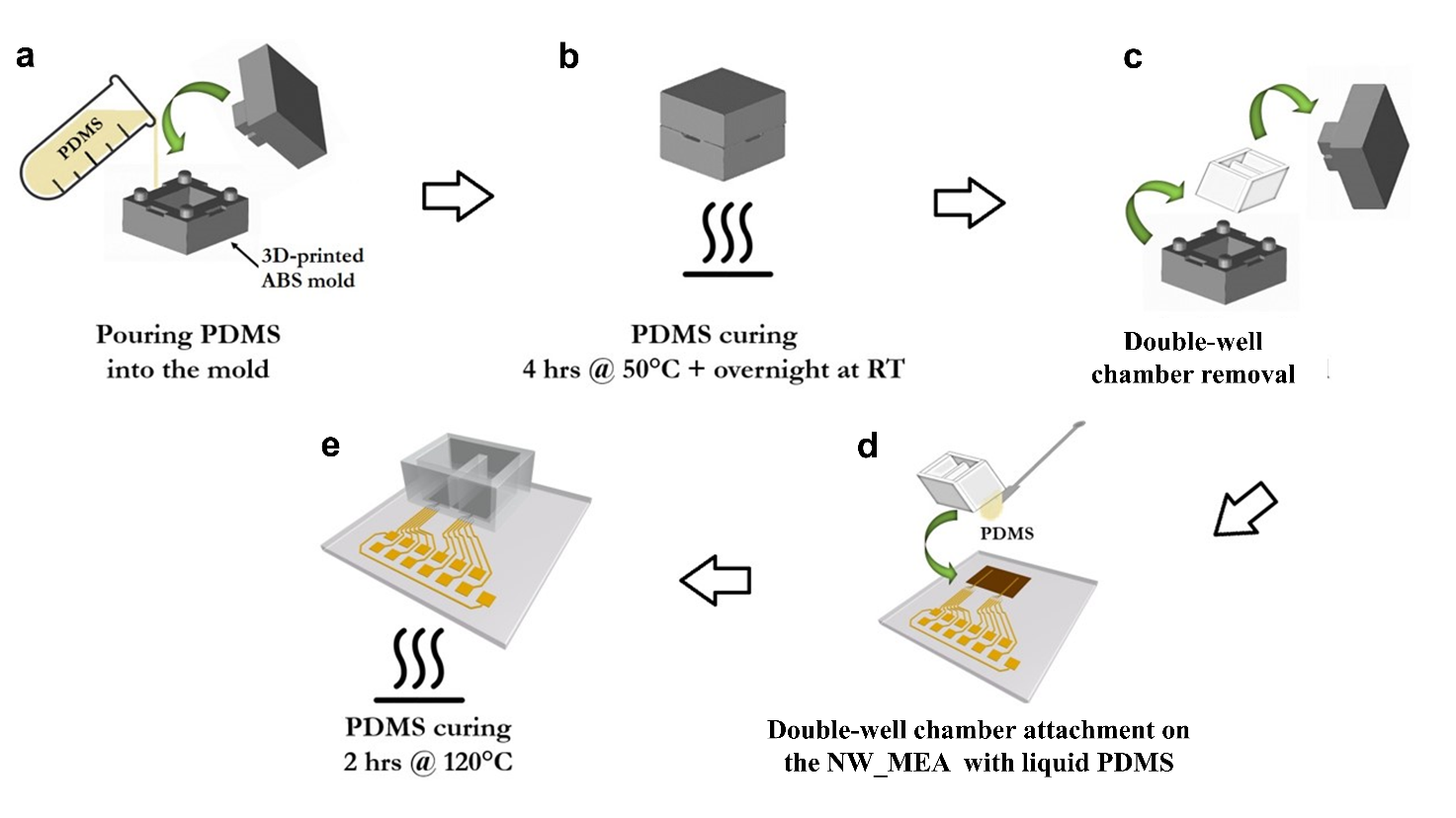


**Figure S8:** **Manufacturing process of the double-well PDMS chamber**. **a**, Pouring a mixture of PDMS (liquid) and crosslinking agent (to cure it) into a 3D printed mold. **b**, Heating at 50°C for 4 hrs and then resting overnight at RT. **c,** Release of the double-well chamber. **d**, Sticking the PDMS double-well chamber onto the NW_ MEA with liquid PDMS. **e,** Curing at 120 °C for 2 hrs


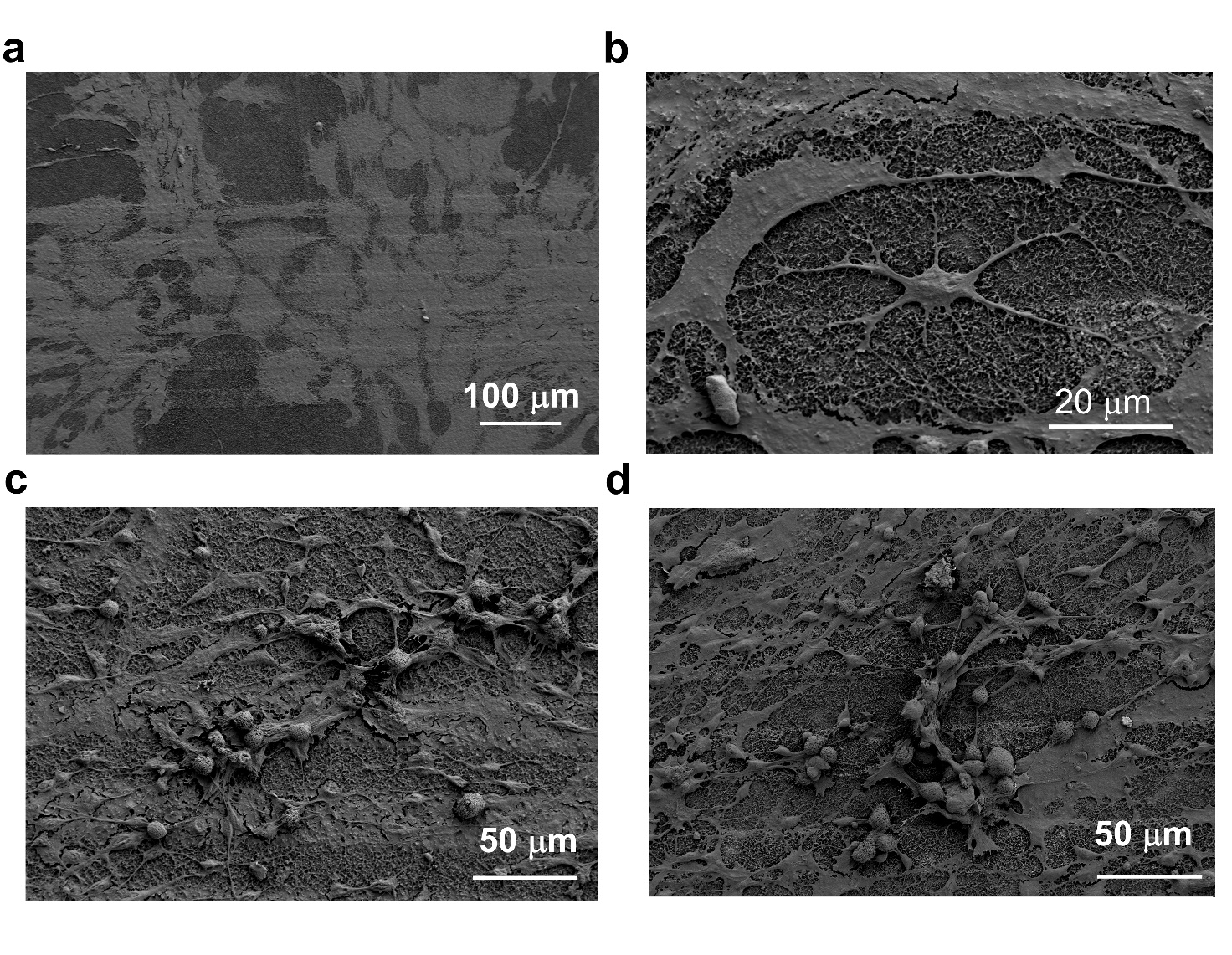


**Figure S9:** **Astrocytes monoculture and astrocytes/DRG cell culture morphology on NW_MEA. a**,**b** SEM images of astrocytes in U1 compartment. **c**, **d** SEM images of DRG neurons and glial cells plated on astrocytes’ layer in U2 compartment.

**Note S2: Comparison of R.M.S. noise amplitude in NW_MEA with and without cells**

In the paper we outlined that the R.M.S. amplitude voltage noise values from electrodes covered solely by astrocytes and the co-culture of astrocytes and DRG neurons-glial cells were 270 µV and 280 µV, respectively. These R.M.S. values were approximately 5 times larger than that of about 50 µV obtained from recordings with the NW_MEA prior to cell culturing. These preliminary recordings were performed using the same pharmacological drug addition protocol as in the cell experiments to test the stability of the device under the addition of different drugs (Figure S10). The increase in R.M.S. amplitude after cell plating is attributed to cell adherence to the electrodes. In addition, the similar R.M.S. values obtained from electrodes covered solely by astrocytes and those covered by astrocytes and DRG neurons-glial cells, indicate that baseline noise primarily originates from the astrocyte layer due to their larger cell size compared to DRG neurons and glial cells.


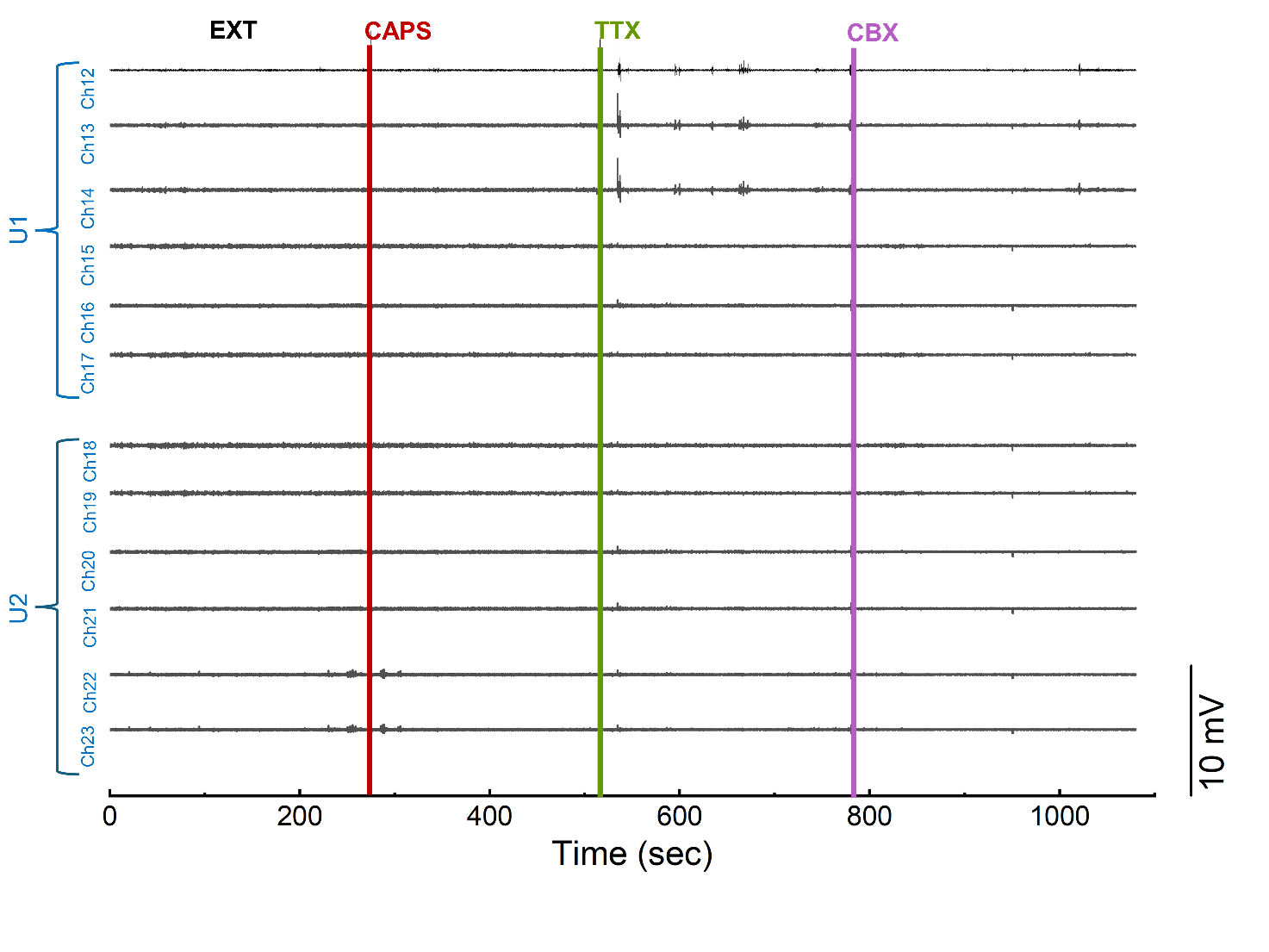


**Figure S10: NW_MEA recordings without cells.** Recordings performed using the NW_MEA without cell in EXT solution by following the pharmacological drug addition protocol used for the cellular signal recordings. No significative signals are visible across all the electrodes. The calculated R.M.S. amplitude of voltage noise was approximately 50 μV, averaged across the 12 electrodes for the whole duration of the recording.

**
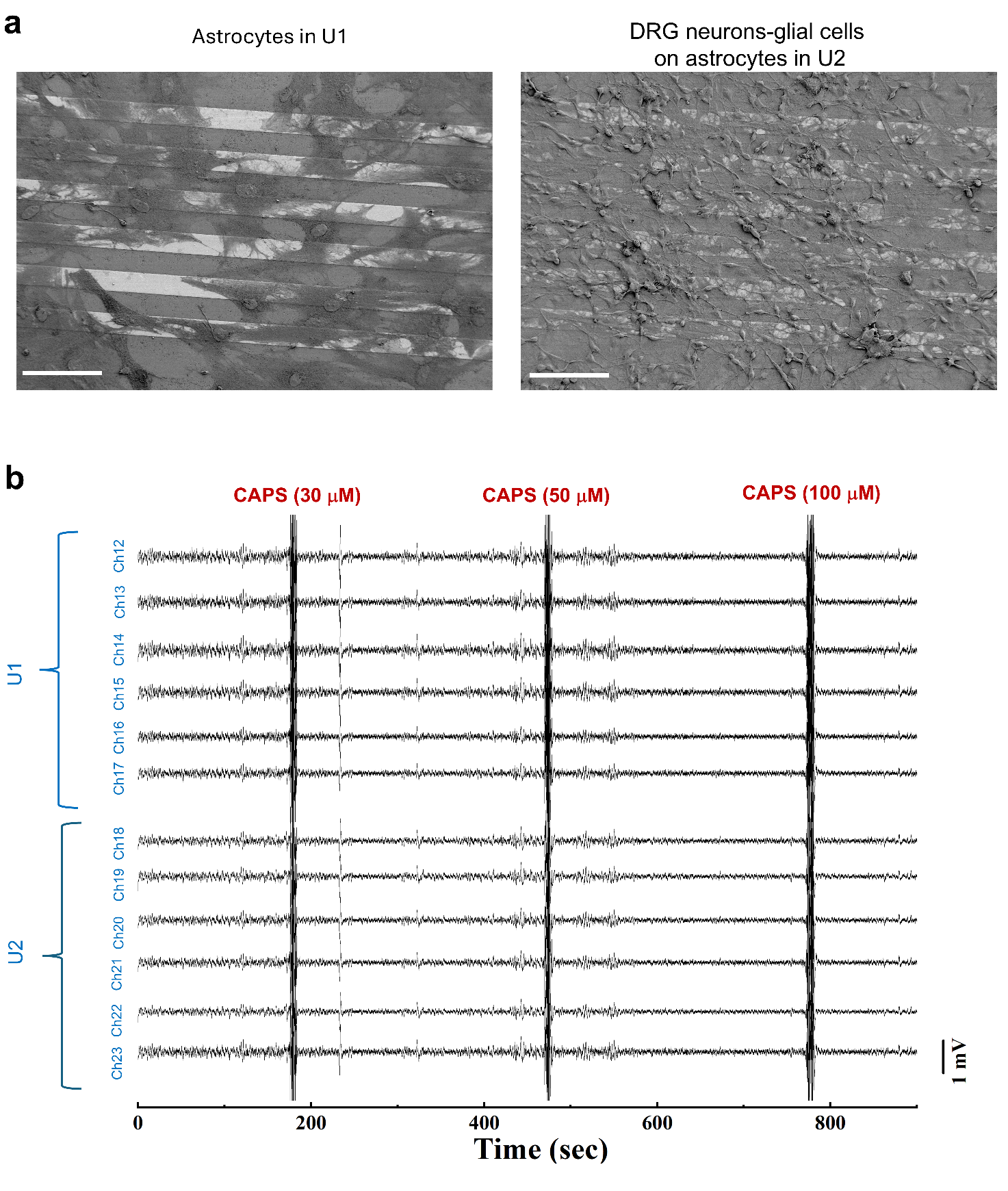
**

**Figure** **S11. Bioelectrical activity recordings** **in** **astrocyte/DRG neuron-glial cell culture using a planar MEA under pharmacological interrogation with CAPS.** Control experiments using planar Ti/Au electrodes on glass slides, designed identically to the NW_MEA**. a**, SEM images of the astrocyte monoculture (left panel), and of DRG neurons and glial cells (right panel) plated on astrocytes in U1 and U2 compartments, respectively, of the planar MEA (scale bar:100 μm). **b,** Voltage vs. time traces recorded from the twelve electrodes in U1 (C12-C17) and U2 (C18-C23). Periods of activity detected under pharmacological stimulation: baseline in EXT solution only, followed by responses to increasing concentrations of CAPS [30 µM], CAPS [50 µM], and CAPS [100 µM]. The increasing CAPS concentrations were tested to determine whether enough amount could elicit a neuronal response. However, no evident bioelectrical activity was recorded, unlike in the case of the NW_MEA.

**Reference**

[1] Nedergaard M, Ransom B, Goldman SA. New roles for astrocytes: redefining the functional architecture of the brain. *Trends Neurosci*. **26**, 523 (2003).

[2] Nascimento, A.I., Mar, F.M. & Sousa, M.M. The intriguing nature of dorsal root ganglion neurons: Linking structure with polarity and function. *Prog Neurobiol*. **168**, 86 (2018).

[3] Nathanniel, P.W.L., Blair, N.T., & Bean B.P. Roles of tetrodotoxin (TTX)-sensitive Na+ current, TTX-resistant Na^+^ current, and Ca^2+^ current in the action potentials of nociceptive sensory neurons. *J Neurosci*. **22**, 10277 (2002).

[4] Amir, R.; Michaelis, M.; Devor M. Membrane potential oscillations in dorsal root ganglion neurons: role in normal electrogenesis and neuropathic pain. *J Neurosci.* **19**, 8589 (1999).

[5] Su, H. Different sensitivity of action potential generation to the rate of depolarization in vagal afferent A-fiber versus C-fiber neurons. *J Neurophysiol.* **125**, 5 (2021).

[6] Tigerholm, J. C-Fiber Recovery Cycle Supernormality Depends on Ion Concentration and Ion Channel Permeability. *Biophys. J.* **108**, 1057 (2015).

[7] Harper, A. A. & Lawson, S. N. Electrical properties of rat dorsal root ganglion neurones with different peripheral nerve conduction velocities. *J. Physiol.* **359**, 47 (1985).

[8] Vasylyev, D.V. & Waxman, S.G. Membrane properties and electrogenesis in the distal axons of small dorsal root ganglion neurons in vitro. *J Neurophysiol.* **108**, 729 (2012
